# Quantifying dynamic pro-inflammatory gene expression and heterogeneity in single macrophage cells

**DOI:** 10.1016/j.jbc.2023.105230

**Published:** 2023-09-09

**Authors:** Beverly Naigles, Avaneesh V. Narla, Jan Soroczynski, Lev S. Tsimring, Nan Hao

**Affiliations:** 1 Department of Molecular Biology, University of California San Diego, La Jolla, California, USA; 2 Department of Physics, University of California San Diego, La Jolla, California, USA; 3 Laboratory of Genome Architecture and Dynamics, The Rockefeller University, New York, New York, USA; 4 Synthetic Biology Institute, University of California San Diego, La Jolla, California, USA; 5 Department of Bioengineering, University of California San Diego, La Jolla, California, USA

**Keywords:** macrophage, single-cell analysis, fluorescence imaging, quantitative imaging, mathematical modeling, gene expression dynamics, pro-inflammatory genes, heterogeneity

## Abstract

Macrophages must respond appropriately to pathogens and other pro-inflammatory stimuli in order to perform their roles in fighting infection. One way in which inflammatory stimuli can vary is in their dynamics—that is, the amplitude and duration of stimulus experienced by the cell. In this study, we performed long-term live cell imaging in a microfluidic device to investigate how the pro-inflammatory genes IRF1, CXCL10, and CXCL9 respond to dynamic interferon-gamma (IFNγ) stimulation. We found that IRF1 responds to low concentration or short duration IFNγ stimulation, whereas CXCL10 and CXCL9 require longer or higherconcentration stimulation to be expressed. We also investigated the heterogeneity in the expression of each gene and found that CXCL10 and CXCL9 have substantial cell-to-cell variability. In particular, the expression of CXCL10 appears to be largely stochastic with a subpopulation of nonresponding cells across all the stimulation conditions tested. We developed both deterministic and stochastic models for the expression of each gene. Our modeling analysis revealed that the heterogeneity in CXCL10 can be attributed to a slow chromatin-opening step that is on a similar timescale to that of adaptation of the upstream signal. In this way, CXCL10 expression in individual cells can remain stochastic in response to each pulse of repeated stimulation, which we also validated by experiments. Together, we conclude that pro-inflammatory genes in the same signaling pathway can respond to dynamic IFNγ stimulus with very different response features and that upstream signal adaptation can contribute to shaping heterogeneous gene expression.

Cells respond to many external signals by initiating gene expression programs to elicit appropriate physiological responses. However, even within a clonal population, there can be significant variability in gene expression among individual cells (1, 2, 3, 4). Several mechanisms, including environmental fluctuations, epigenetic regulation, and the inherent stochasticity of biochemical reactions, can potentially contribute to this heterogeneity (5, 6, 7, 8, 9). Importantly, cell-to-cell variability in gene expression can lead to functional consequences for development, disease progression, and response to therapy (10, 11). For example, some cancer cells in a tumor can be more resistant to chemotherapy than others due to differences in the expression of genes involved in drug metabolism or DNA repair (12, 13, 14). Similarly, during hematopoiesis, the high expression heterogeneity of the stem cell marker Sca-1 leads to different fate decisions toward erythroid or myeloid lineages (15). In this study, we focus on heterogeneity in the immune response and investigate the dynamics of gene expression in single macrophage cells.

Macrophages are innate immune cells that perform a diverse range of functions in the body and adapt their functional response to their local signaling environment. Macrophage phenotypes exist along a continuous spectrum and single cells dynamically shift between states (16). Here, we focus on the pro-inflammatory M1 phenotype, wherein macrophages play antimicrobial roles and promote inflammatory immune responses. This phenotype occurs *in vivo* in response to bacterial or viral infections and is modeled *in vitro* by exposure to lipopolysaccharide (LPS) or interferon-gamma (IFNγ) (17).

Macrophages are highly heterogeneous cells, and macrophage heterogeneity has been studied *in vitro* both before and after infection (18, 19, 20). In mouse bone marrow–derived macrophages infected with *Salmonella enterica*, infected macrophages adopt diverse gene expression states with varying levels of pro-inflammatory gene expression (21). In diseases such as tuberculosis, variability in how macrophages respond to infection leads to dramatic differences in clinical outcome (22, 23). This macrophage heterogeneity is also seen *in vivo*, where macrophages have diverse functions and gene expression patterns in many organs, including the lung (24), brain (25), and peritoneum (26), as well as in response to inflammation (27).

Single-cell RNA sequencing has uncovered gene expression heterogeneity among individual macrophages and other myeloid cells exposed to pro-inflammatory signals such as LPS and IFNγ *in vitro* (28, 29, 30, 31). These experiments eliminate the element of bacterial heterogeneity and have shown that heterogeneity is present in aspects of immune responses other than direct interaction with a pathogen. However, these single-cell RNA sequencing studies cannot reveal how single-cell transcriptional patterns vary in time in response to pro-inflammatory stimuli. Bulk studies show that pro-inflammatory gene expression is highly temporally regulated, with sets of earlier primary response genes and later secondary response genes that each share certain elements of their epigenetic and transcriptional regulation (32, 33). However, the intersection of heterogeneity and gene expression timing remains poorly understood.

The primary pathway in which pro-inflammatory gene expression in macrophages has been studied at the single-cell level over time is in genes induced by the transcription factor (TF) NFκB, which translocates to the nucleus upon stimulation with pathogens, pathogen analogs, and certain other pro-inflammatory stimuli. NFκB activates different gene expression programs based on its residence time dynamics in the nucleus, and the residence time dynamics depend on the identity of the upstream signal and can vary between clonal cells (34, 35, 36, 37). Varied TF dynamics leading to differential gene expression and cellular outcome have also been seen in other systems such as the yeast Msn2 system and p53 expression in the MCF7 breast cancer cell line (38, 39, 40).

These studies used quantitative analysis of single-cell time traces coupled with mathematical modeling to uncover the gene expression networks and mechanisms that encode and decode complex environmental signals, since bulk measurements of dynamical behavior can distort individual patterns due to averaging over different single cells (39). Observing gene expression output in response to dynamic upstream signals can reveal elements of network structure, whether the dynamic upstream signal is natural (*e.g.*, extracellular signal-regulated kinase signaling in response to epidermal growth factor versus nerve growth factor (41)) and/or is exogenously applied (38, 42, 43). Mathematical modeling can rule in or out possible underlying network motifs and mechanisms of epigenetic regulation of gene expression, as well as suggesting testable perturbations that will change the dynamical behavior of the system (42, 44).

Macrophage signal processing and heterogeneity is essential for properly modulating immune responses both at initial pathogen recognition and within the later stages of the innate and adaptive immune response; however, signal processing in the later stages of the innate and adaptive immune response is poorly studied. To this end, we focused on the cytokine IFNγ, which polarizes macrophages to an M1 phenotype but is secreted by other immune cells rather than being directly associated with a pathogen. Macrophages encounter IFNγ in various temporal patterns during the immune response as it is secreted by natural killer cells during the innate immune response and CD4 Th1 cells during the adaptive immune response (45, 46). IFNγ binds to the IFNγ receptor and signals through the JAK-STAT signaling pathway, leading to STAT1 phosphorylation, homodimerization, and translocation into the nucleus where the phosphorylated STAT1 homodimer acts as a TF for the downstream gene expression program (45). Misregulation of IFNγ signaling is pervasive in disease, with too little IFNγ leading to poor infection response, too much IFNγ leading to excessive inflammation and autoimmune diseases, and dysregulation of IFNγ being important in tumor immunology (47, 48, 49).

In this study, we investigate the single-cell expression dynamics of the genes IRF1, CXCL10, and CXCL9, which are all induced by STAT1 in response to IFNγ (50, 51, 52). IRF1 and CXCL10 are representative primary response genes with different kinetics (early for IRF1, later for CXCL10), and CXCL9 is a secondary response gene (52). IRF1 encodes a TF important for the expression of many downstream pro-inflammatory genes (52, 53, 54, 55). CXCL10 and CXCL9 encode chemokines that recruit T cells and other cells expressing the CXC chemokine receptor 3 to the site of inflammation and have been implicated in a number of disease conditions (56, 57, 58). As an example, CXCL10 expression is important early in SARS-CoV-2 infection to create an antiviral environment, but later in infection, high CXCL10 and CXCL9 contribute to the cytokine storm that leads to severe disease (59, 60).

## Results

### Quadruple-reporter macrophage cell lines to study IFNγ-induced gene expression

**Figure 1:**
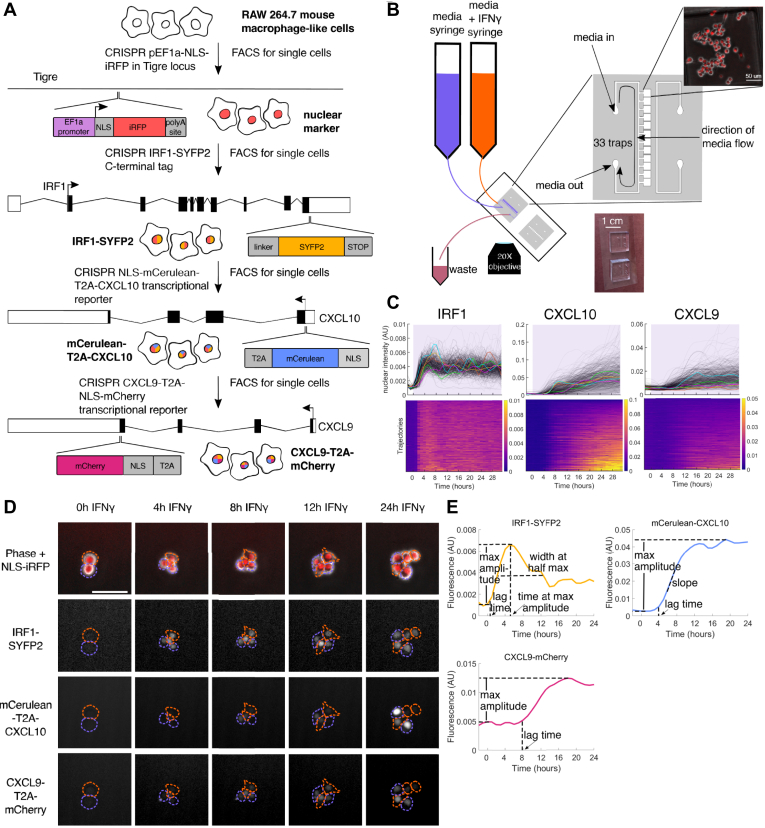
**Endogenous CRISPR knock-in cells for measuring IFNγ-induced gene expression in single macrophage cells over time.***A*, schematic of cell line construction. *B*, diagram of microfluidic chip and setup used in these experiments. *C*, IRF1, CXCL10, and CXCL9 gene expression responses to 10 ng/ml IFNγ in a 24-well plate. In the top row, each *gray line* is a cell, with five traces colored as examples. The bottom row shows the same data as heatmaps with each row representing one cell. The heatmaps are sorted by the maximum fluorescence value for each cell for each gene, resulting in the sort order being different for each of the three genes. IFNγ is added at time 0. Color in the heatmaps represents gene expression fluorescence. *Purple* shading indicates time when the cells are exposed to IFNγ. *D*, sample images of cells experiencing IFNγ stimulation. Single cells are outlined over time, with the same color outline showing the same cell and its offspring. Scale bar on top left image represents 50 μm. *E*, schematic of sample traces for IRF1, CXCL10, and CXCL9 showing the features extracted from each trace. IFNγ, interferon-gamma.

We used CRISPR/Cas9 genome editing to create a RAW 264.7 mouse macrophage-like cell line that expressed endogenous fluorescent reporters for each of IRF1, CXCL10, and CXCL9 in the same single cells, as well as a nuclear marker for use in image analysis (Fig. 1*A* and Experimental procedures). RAW 264.7 cells have been used as a model for much of the work studying NFκB signaling dynamics in macrophages (19, 34, 61, 62, 63, 64), gene regulation in pro-inflammatory macrophages (65, 66, 67), and *in vitro* macrophage models for mycobacterial infection (68, 69). The cell line created in our study is heterozygous for the SYFP2 tag at the IRF1 locus and homozygous for both the mCerulean knock-in at the CXCL10 locus and the mCherry knock-in at the CXCL9 locus. For CXCL10 and CXCL9, the fluorescent protein DNA sequence is connected to the endogenous chemokine DNA sequence by a T2A translational skip site, ensuring that the endogenous chemokine can be secreted normally and the fluorescent reporter protein attached to a nuclear localization signal (NLS) is retained in the nucleus where it can be measured (Fig. 1*A*) (70, 71). A list of all cell lines created in this work can be found in Table S1. To confirm that STAT1 signaling is not perturbed in our quadruple knock-in cell line, we have shown using quantitative reverse transcription polymerase chain reaction (RT-qPCR) that the expression level of IRF1, a gene immediately downstream of STAT1, is unchanged between untagged RAW 264.7 cells and the quadruple knock-in cell line created here (Fig. S1*A*).

We then exposed these cells to IFNγ in either a 24-well plate or a microfluidic device for 31 h, taking fluorescence images every hour. We used an image-processing pipeline to extract fluorescence measurements for each gene over time in single cells (Experimental procedures). The microfluidic device allows us to expose the cells to time-variant stimulus patterns and have media turnover in less than 15 min (Fig. 1*B*) (72). Sample single-cell traces are shown in Figure 1*C* and a sample field of view in Figure 1*D*, where we can see substantial heterogeneity in CXCL10 and CXCL9 expression between cells. A sample movie for cells experiencing 10 ng/mL IFNγ can be seen in Supplemental Movie 1.

We can extract several features from these traces that together describe the response for each gene (Fig. 1*E*). We are also interested in how these features correlate with each other and change in response to dynamic stimulation, which we will describe below. For each gene, we measure the lag time before protein fluorescence can be measured, as well as the maximum expression amplitude (Fig. S1, *K*, *L*, *P*, *Q*, *U* and *V*). Additionally, for CXCL10, we measure the slope of the response and for IRF1, the width of the peak and the time at the maximum amplitude (Fig. S1, *M*, *N* and *R*).

To confirm that IFNγ does not induce global changes in gene expression, we measured nuclear marker expression upon IFNγ stimulation and saw that its levels remain constant (Fig. S1*C*). We also confirmed that the pre-stimulus expression levels of IRF1, CXCL10, and CXCL9 do not correlate with their eventual maximum fluorescence levels (Fig. S1, *D–G*). To determine if there is correlation between these genes on a single-cell level, we correlated maximal expression levels of IRF1, CXCL10, and CXCL9 and see no correlation between IRF1 and either CXCL10 or CXCL9 and a weak positive correlation between CXCL10 and CXCL9 (Fig. S1, *B* and *H–J*). When we cross-correlate features other than amplitude of different genes in the same cells, we see no strong correlations (Fig. S1, *O*, *S*, *T*, *W* and *X*). Based on these data, we will treat each gene independently in our analysis.

### IRF1, CXCL10, and CXCL9 are expressed differently in response to IFNγ stimulation of varying concentration or duration

**Figure 2:**
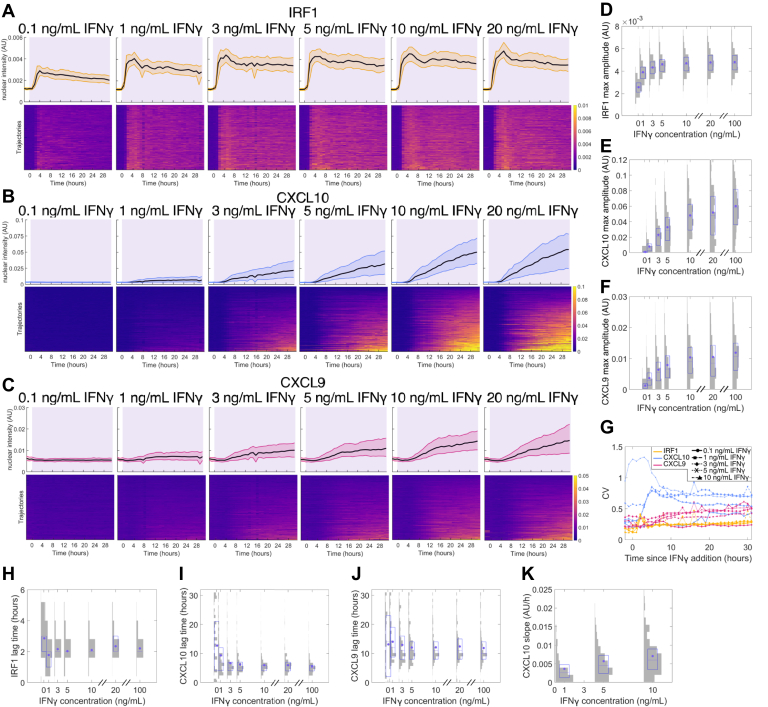
**IRF1, CXCL10, and CXCL9 response to varying concentrations of IFNγ stimulation.***A–C*, gene expression responses for 0.1 to 20 ng/ml IFNγ in a 24-well plate with IFNγ added at 0 h. The top row of plots for each gene has a *black line* at the median and shading to the 25th and 75th percentiles, and each row in the heatmaps in the bottom row represents one cell. Each heatmap is sorted vertically by single-cell maximum value for that specific gene. *Purple* shading indicates when the cells are exposed to IFNγ. Color in the heatmaps represents gene expression amplitude. *A*, is IRF1, *B* is CXCL10, *C* is CXCL9. *D–F*, histograms overlaid with box plots showing maximum IRF1 (*D*), CXCL10 (*E*), and CXCL9 (*F*) expression in single cells for each IFNγ concentration. *G*, coefficient of variation of expression at each timepoint (see Experimental procedures) for all genes across IFNγ concentrations. *H–J*, histograms overlaid with box plots showing the lag time for IRF1 (*H*), CXCL10 (*I*), and CXCL9 (*J*) in single cells for each IFNγ concentration. *K*, histograms overlaid with box plots showing the CXCL10 slope in single cells for each IFNγ concentration. For all box plots, the *purple* dot is the mean, the *purple* center line is the median, and the *purple* box is the 25th-75th percentile. The *gray* shading shows the histogram distribution among single cells for each condition. IFNγ, interferon-gamma.

To investigate how these gene expression features change in response to dynamic stimulation, we first exposed the cells to constant IFNγ stimulation of different concentrations. IRF1 shows fast and uniform expression in all cells, with the cells responding even to 0.1 ng/ml IFNγ and saturating at 3 ng/ml IFNγ. (Fig. 2, *A* and *D*). When tissue IFNγ levels have been measured after infection, they have ranged from 0.1 ng/ml to 10 ng/ml, showing that this is a physiologically relevant range (73, 74). The width and timing of the IRF1 peak do not vary with the concentration of IFNγ (Fig. S2, *C* and *D*). At all concentrations, we see that the majority of cells reach their maximal expression around 4 - 6 h after onset of stimulation and have a peak width around 9 h, with a minority of cells peaking later (Fig. 2, *C* and *D*). At a single-cell level, neither peak time nor width correlates with expression amplitude (Fig. S2, *H* and *I*). However, we note that peak width correlates positively with peak time. This is explained by the fact that later peaks are due to IRF1 expression staying high after the initial rise, resulting in a wider peak (Fig. S2*J*).

In contrast, CXCL10 shows slower and more heterogeneous expression, which saturates at 10 ng/ml IFNγ and has a sharp increase in amplitude from 0.1 ng/ml to 10 ng/ml (Fig. 2, *B* and *E*). CXCL10 lag time does not vary with IFNγ concentration and has a narrow and symmetric distribution (Fig. 2*I*), indicating that cells begin to express CXCL10 at a defined time regardless of IFNγ concentration. CXCL10 lag time also does not correlate with gene expression amplitude on a single-cell level (Fig. S2*K*). In contrast, on a population level, CXCL10 slope increases with IFNγ concentration up to 10 ng/ml, and in single cells the slope correlates positively with CXCL10 expression amplitude (Figs. 2*K* and S2*L*). Intriguingly, we observe that the proportion of cells that express high levels of CXCL10 increases with increasing IFNγ concentration, but at all concentrations there is always a fraction of cells with very low or no CXCL10 expression (Fig. S2*N*). The proportion of these non-responding cells remains unchanged even when IFNγ concentration is increased beyond saturation (10–100 ng/ml) (Figs. 2*B* and S2, *B* and *N*). Similarly to CXCL10, CXCL9 expression increases nonlinearly in response to IFNγ doses from 0.1 ng/ml up to 10 ng/ml (Fig. 2*F*), shows substantial heterogeneity (Fig. 2*C*), and has a lag time that remains unchanged across IFNγ concentrations and is uncorrelated with expression amplitude in single cells (Figs. 2*J* and S2*M*). However, we note that the lag time distribution for CXCL9 is much wider than for IRF1 or CXCL10.

**Figure 3:**
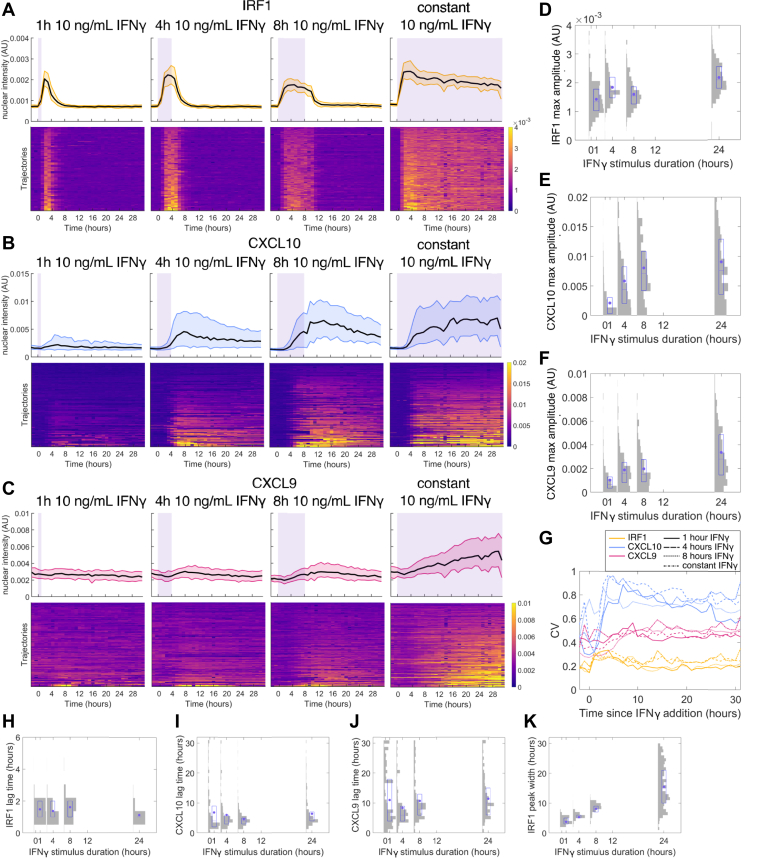
**IRF1, CXCL10, and CXCL9 response to varying durations of IFNγ stimulation.***A–C*, gene expression responses for 1 h, 4 h, 8 h, and constant 10 ng/ml IFNγ stimulation in a microfluidic device with IFNγ added at 0 h. The *top* row of plots for each gene has a *black line* at the median and shading to the 25th and 75th percentiles, and each row in the heatmap represents one cell. Each heatmap is sorted by single-cell maximum value for that specific gene. *Purple* shading indicates the time during which the cells are exposed to IFNγ. Color in the heatmap corresponds to gene expression amplitude. *A*, is IRF1, *B* is CXCL10, *C* is CXCL9. *D–F*, histograms overlaid with box plots showing maximum IRF1 (*D*), CXCL10 (*E*), and CXCL9 (*F*) expression in single cells for each IFNγ duration. *G*, coefficient of variation of expression at each timepoint (see Experimental procedures) for all genes across IFNγ durations. *H–J*, histograms overlaid with box plots showing the lag time for IRF1 (*H*), CXCL10 (*I*), and CXCL9 (*J*) in single cells for each IFNγ duration. *K*, histograms overlaid with box plots showing the IRF1 peak width in single cells for each IFNγ duration. For all histograms overlaid with box plots, the *purple* dot is the mean, the center *purple* line is the median, and the *purple* box is the 25th-75th percentile. *Gray* shading shows the histogram distribution among single cells for each condition. IFNγ, interferon-gamma.

We next exposed the cells to 1-h, 4-h, 8-h, or constant durations of 10 ng/ml IFNγ stimulation in a microfluidic device. We chose 10 ng/ml because it is the saturating concentration in our system. Cells show a homogeneous IRF1 peak with a similar amplitude and lag time across all IFNγ durations (Fig. 3, *A*, *D* and *H*), while the width of the peak increases with IFNγ duration until it saturates between 8 and 24 h (Fig. 3, *A* and *K*). For CXCL10, increasing IFNγ duration leads nonlinearly to increased gene expression, with 1 h of IFNγ inducing very low expression and expression amplitude saturating between 4 h and 8 h of IFNγ stimulation (Fig. 3, *B* and *E*). The CXCL10 slope is also lower with shorter IFNγ duration but saturates at around 4 h of stimulation (Fig. S3*B*). The CXCL10 lag time is similar for all IFNγ durations of 4 h or longer, while for 1 h of stimulation, the cells that do express CXCL10 have a very short lag time, showing that this 1-h stimulation only induces the earliest cells (Fig. 3*I*). Increasing IFNγ duration also increases the proportion of cells that strongly express CXCL10, but there remains a fraction of cells with very low CXCL10 expression even in response to constant stimulation (Fig. 3*B* and S3*G*). CXCL9 expression increases with IFNγ duration but does not saturate by 24 h; the cells continue to produce CXCL9 for as long as stimulus is provided (Fig. 3, *C* and *F*).

Taken together, these results demonstrate that IRF1 expression is fast and uniform among cells, whereas CXCL10 expression is slower and highly heterogeneous, and CXCL9 expression is even slower than CXCL10 and also heterogeneous. IRF1 responds strongly to low-concentration or short-duration IFNγ stimulation, while CXCL10 and CXCL9 act as high-pass filters to filter out these lower stimuli and respond strongly only to higher-concentration or longer-duration stimulation. We see that lag time is invariant to dynamic stimulation (as would be expected as cells have no knowledge of future events) and that the lag time distributions for IRF1 and CXCL10 are narrow while the distributions for CXCL9 are wider (Fig. 2, *H*–*J*). This shows that heterogeneity in CXCL10 expression comes from expression amplitude rather than timing but that CXCL9 expression also varies in timing. Other than amplitude, the features that vary between cells or with dynamic input are CXCL10 slope, which increases with IFNγ concentration, and IRF1 peak width, which increases with IFNγ duration. We see no identifiable spatial pattern in the CXCL10 and CXCL9 heterogeneity.

In addition, for CXCL10, increasing either input amplitude or duration can increase the proportion of cells with high expression levels of the gene; however, even upon constant stimulation with saturating concentrations of IFNγ, there remains a substantial fraction of cells with low-to-no expression. To quantify cell-to-cell variation in gene expression, we calculated the coefficient of variation (CV) for each gene (see Experimental procedures) and observed that CXCL10 has the highest CVs and IRF1 has the lowest under all the stimulation conditions tested (Figs. 2*G* and 3*G*). The CV for all genes varies minimally across IFNγ concentrations and durations, despite the fact that the mean expression level of CXCL10 is altered by up to 6-fold (Figs. 2*G* and 3*G*).

### Computational modeling of gene expression responses to dynamic stimulation

**Figure 4:**
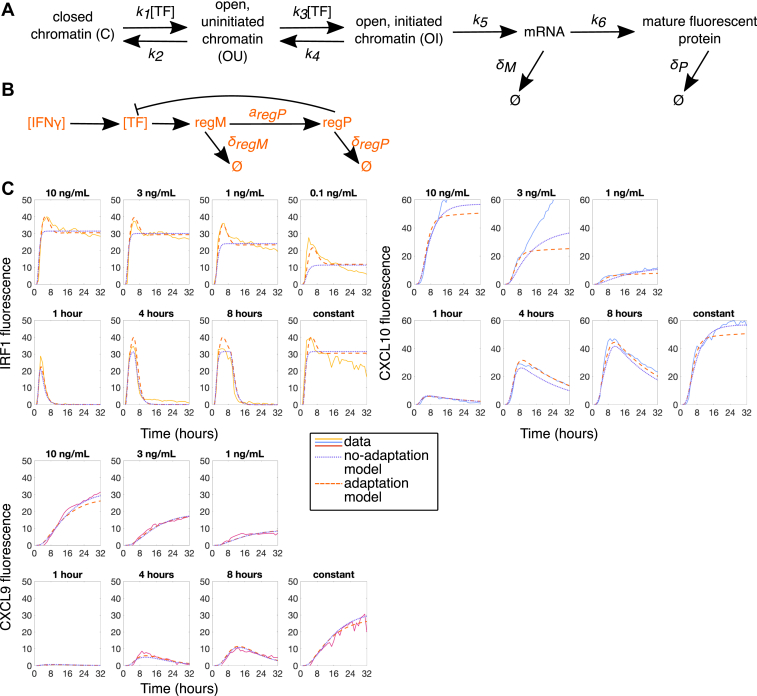
**Deterministic modeling of IFNγ-induced gene expression.***A*, schematic diagram of the gene expression model architecture with 3-state chromatin dynamics and gene transcription and translation for all three genes of interest (IRF1, CXCL10, and CXCL9). *B*, transcription factor (TF) adaptation model. *C*, ODE model without adaptation (*purple dotted lines*) and with adaptation (*orange dashed lines*) best fit to IRF1, CXCL10, and CXCL9 population mean data (*yellow*, *blue*, or *pink solid lines*) in all IFNγ concentration and duration conditions. Prior to fitting, the fluorescence data here were rescaled to peak at the same level (about 40) for each gene. Duration experiments were done with 10 ng/ml IFNγ and concentration experiments have constant IFNγ. IFNγ, interferon-gamma; ODE, ordinary differential equation.

We found two features of our dynamic stimulation data particularly striking: the different ways in which each gene either filtered out or responded to IFNγ stimulation of low amplitude or short duration and the persistence of cells with low-to-no CXCL10 expression in all conditions. To investigate the possible mechanisms of these phenomena, we constructed deterministic and stochastic models of this signaling and gene expression system. We began with an ordinary differential equation (ODE) model that describes chromatin opening, transcription initiation, mRNA synthesis, mRNA translation, and fluorescent protein maturation, as well as degradation of the mRNA and protein (Fig. 4*A*). We fit this model to the mean protein time courses in response to IFNγ stimulation of all concentrations and durations for each of IRF1, CXCL10, and CXCL9 (Fig. 4*C*, Table S2 for best fit parameters, Experimental procedures for detailed description of modeling). For CXCL10, we fit only to the first 12 h of the 24-well plate (concentration) experiments because we see that after 12 h, the CXCL10 response in a microfluidic device (used in the duration experiments) stops increasing, but in the plate it continues to increase. This is likely because the cells are secreting a paracrine effector that leads to increased CXCL10 expression over time in the plate experiments but is washed out in the microfluidic experiments. As we are not trying to model paracrine effectors, we chose to ignore this region in the plate experiments. When we compare the parameters and look at the intermediate model states, we see that in our model chromatin opening and transcription initiation are much slower for CXCL10 than for IRF1 and slower for CXCL9 than CXCL10 (Table S2 and Fig. S4*A*).

**Figure 5:**
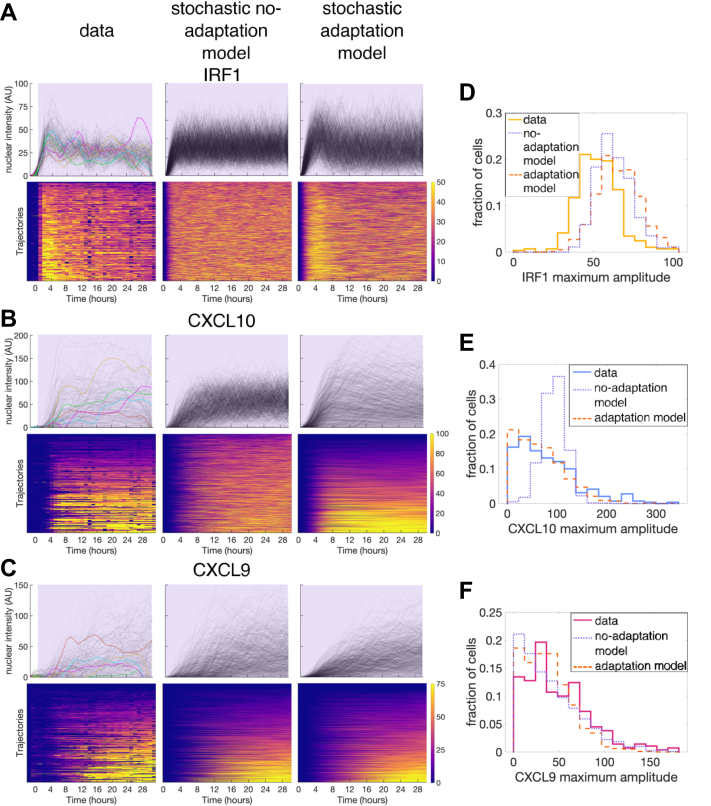
**Stochastic modeling of IFNγ-induced gene expression.***A–C*, comparison of experimental and stochastic simulation results for both no-adaptation and adaptation models for constant 10 ng/ml IFNγ stimulus for IRF1 (*A*), CXCL10 (*B*), and CXCL9 (*C*). The *top* row panels show the fluorescence of each cell as a *gray* line and the *bottom* row panels show the same data in a heatmap. Heatmaps are sorted by maximum value for each gene individually. *Purple* shading indicates when the cells (simulated or experimental) are exposed to IFNγ. *D–F*, histograms showing distribution of IRF1 (*D*), CXCL10 (*E*), and CXCL9 (*F*) maximum amplitude both experimentally (*solid line*) and in the no-adaptation (*purple dotted line*) and adaptation (*orange dashed line*) stochastic models for constant stimulation of 10 ng/ml IFNγ. IFNγ, interferon-gamma.

While this deterministic model reasonably describes the mean response of the cells, it cannot capture the cell-to-cell variability that we see in our data. We were particularly interested in the striking heterogeneity in CXCL10 expression, especially given its relatively early expression following IFNγ stimulation. Our hypothesis was that this CXCL10 variability may be due to slow and strongly stochastic chromatin dynamics. To test this hypothesis, we developed a stochastic model to model the dynamics of individual cells and characterize the distribution of responses. We used the same species and reactions as in our deterministic model but treated them stochastically using the direct Gillespie algorithm (75) with the same rates as in our fitted deterministic model. While we did observe variability in cellular CXCL10 time traces among individual runs in our simulations, we also saw that, given long enough stimulation time, all cells eventually express CXCL10 within the timeframe of our experiment (Fig. 5*B*, middle column). This results in a narrow distribution of maximal CXCL10 expression levels in the population of cells, contrary to what we see in experiments where even for continuous stimulation there is a significant fraction of cells that never express CXCL10 (Fig. 5*B* first column and Fig. 5*E*).

There could be two explanations for this discrepancy. One possible explanation is that there is a stable subpopulation of nonresponding cells that have CXCL10 permanently silenced at the chromatin level and thus never express CXCL10. The other possible explanation is that there is adaptation of the upstream signaling pathway so that after a certain time the stimulation of downstream gene expression gets suppressed. Thus, even for persistent upstream IFNγ stimulation, cells only have a finite time window in which they can express CXCL10 in response to this stimulation. Previous studies have shown that constant IFNγ stimulation leads to a peak of STAT1 nuclear translocation around 0.5-1 h after stimulation, followed by STAT1 slowly leaving the nucleus, and that STAT1 nuclear localization is essential for its TF activity (45, 76). We have confirmed these STAT1 dynamics in our system using immunofluorescence for STAT1 (Fig. S4*B*). We have further experimental evidence for adaptation in our IRF1 data, where we see that upon constant IFNγ stimulation, IRF1 levels decrease from their peak at a rate much slower than their putative degradation rate and in fact IRF1 remains expressed at a submaximal level for as long as stimulus is present. This can best be explained by continued production of IRF1 at a substantial but submaximal rate due to upstream signal adaptation (Fig. 4*C*, top row of IRF1 data).

To account for this adaptation of the upstream signal, we modified our stochastic model to include a STAT1-induced negative regulator that inhibits STAT1 in a negative feedback manner (Fig. 4*B* and S4*A* TF trace). This modification sets an upper limit on the time by which CXCL10 must be expressed in order to be expressed at all, and this results in the simulated single-cell amplitude distribution more closely matching the data (Fig. 5*E*). Adaptation improves the stochastic model fit to the data for CXCL10 and CXCL9 lag time as well (Fig. S5, *B* and *C*). We also incorporated this stimulus adaptation back into our deterministic model and observed that this greatly improved the agreement between the model and the data, especially for IRF1 under constant stimulation (Fig. 4*C*). When we use the stochastic model with adaptation to compute the distribution of both the maximum amplitude and lag time across all concentration and duration conditions, we see that the histograms match the experimental data for most conditions (Fig. S5*D*). From this, we conclude that a combination of the slow chromatin-opening step and the upper limit on the time for chromatin-opening set by the timescale of STAT1 adaptation can produce the protein expression of varying amplitude but defined timing that we see from CXCL10, and that this can also produce the CXCL10 low-to-non-responders that we see even under constant stimulation.

### Response to repeated IFNγ pulses suggests slow, stochastic chromatin opening controls CXCL10 gene expression

While our model with adaptation describes the data for persistent and single-pulse stimulation quite well, the data reported thus far cannot eliminate the possibility that CXCL10 is simply epigenetically silenced in the low-to-nonresponding cells. We also cannot rule out cell cycle effects, as previous studies have revealed that there are genes whose expression is biased towards specific cell cycles stages and thus the cell cycle could be a source of gene expression variability (2, 77, 78, 79, 80, 81). To assess these possibilities, we performed further experiments and analyses. To evaluate the dependence of CXCL10 expression on cell cycle progression, we grouped cells by approximate cell cycle stage at the onset of IFNγ stimulation and observed that cells in all stages of the cell cycle respond similarly (Fig. S6, *A* and *B*), thus ruling out the cell cycle as a driver of the observed heterogeneity.

**Figure 6:**
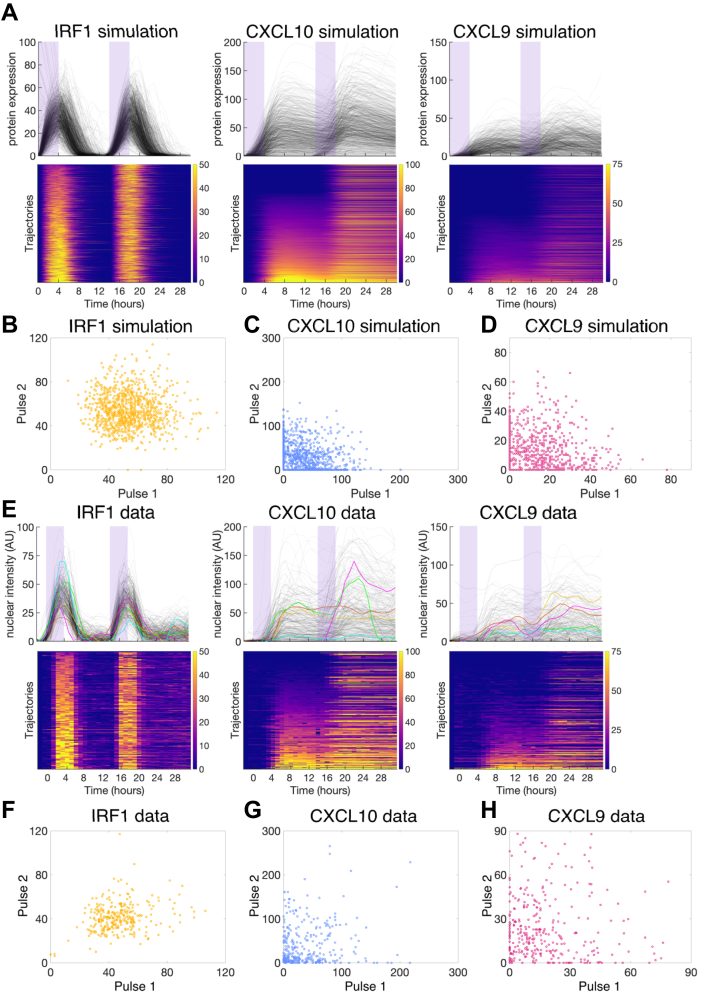
**Model simulation and experimental results for cells exposed to two pulses of IFNγ stimulation.***A*, stochastic adaptation model results of 1000 simulated cells exposed to two 4-h pulses of 10 ng/ml IFNγ with a 10-h off period between the pulses. Line plots show each cell as a line and heatmaps show each cell as a row. Heatmaps are sorted by maximum fluorescence in the first 14 h after the first onset of stimulation. *Purple* shading indicates time when simulated cells are exposed to IFNγ. *B–D*, the protein expression amplitude change that each simulated cell in *A* achieved in each of the two pulse windows (from pulse onset to 9 h after the pulse ended) plotted against each other for IRF1 (*B*), CXCL10 (*C*), and CXCL9 (*D*). *E*, experimental data of cells exposed to two 4-h pulses of 10 ng/ml IFNγ with a 10-h off period between the pulses. Line plots show each cell as a line and heatmaps show each cell as a row. Heatmaps are sorted by maximum fluorescence in the first 14 h after the first onset of stimulation. *Purple* shading indicates time when cells are exposed to IFNγ. *F–H*, the maximum amplitude of expression that each experimental cell in *C* achieved in each pulse window plotted against each other for IRF1 (*F*), CXCL10 (*G*), and CXCL9 (*H*). IFNγ, interferon-gamma.

To test whether CXCL10 was permanently epigenetically silenced in the low-to-nonresponding cells, we decided to expose our cell populations to two pulses of IFNγ separated by a long enough interval so that any possible adaptation would be recovered. If the cells had permanently silenced chromatin, the cells that did not respond to the first pulse would also not respond to the second pulse. However, if the lack of response is due to the stochastic event of chromatin being closed during a pulse, then some cells that are silent during the first pulse could still respond during the second and vice versa. We first tested this scenario in our stochastic model with adaptation, where we simulated stimulating the cells with two 4-h pulses of IFNγ with a 10-h off-interval. Our model predicted that all cells would show uniform expression of IRF1 in response to each input pulse, which is a consequence of fast chromatin dynamics for IRF1 (Fig. 6, *A* and *B*). However, for CXCL10, the chromatin dynamics are much slower, and so some of the cells that did not express CXCL10 in response to the first pulse still expressed CXCL10 in response to the second pulse (Fig. 6, *A* and *C*). This CXCL10 result contrasts with what we would see if CXCL10 is stably silenced, where nonresponding cells would remain nonresponding to both pulses. The model also predicts low CXCL9 expression, as expected for such a short stimulus duration, but that the CXCL9 response to the second pulse will be slightly higher than that to the first pulse given that the chromatin at the CXCL9 locus closes slowly and so the second pulse response is still incorporating the continued response to the first pulse.

We performed the corresponding two-pulse experiment with the same protocol and observed single-cell responses that are quantitatively consistent with our model simulations (Fig. 6, *E*–*H*). These results rule out the possibility that CXCL10 is stably silenced in a subpopulation of cells and support our model, in which CXCL10 expression is controlled by slow, stochastic chromatin opening in response to an input signal that adapts around 4 h, slightly longer than the timescale of chromatin opening. When we categorize the cells as responding to the first, second, both, or neither pulses, the probability of responding to a given pulse is similar regardless of whether the cell responds to the other pulse, further supporting the stochastic nature of gene activation as predicted by our model (Table S4). We also see that the data match the model predictions for IRF1 and CXCL9, specifically that, as predicted by the model, the CXCL9 response to the second pulse is somewhat higher than to the first pulse (Fig. 6, *B*, *D*, *F* and *H*).

We further analyzed the quantitative features of the CXCL10 response to repeated input. To do this, we categorized cells as responding to the first (+/−), second (−/+), both (+/+), or neither (−/−) pulses. When we compare the response to the first pulse of stimulus between +/+ cells and +/− cells, we see that both the amplitude and slope of the first pulse response in +/− cells are higher than that of +/+ cells (Fig. S6, *C* and *D*). When we compare the response to the first and second pulses in +/+ cells, we see that the slope of the second response is higher than that of the first response (Fig. S6*E*). Comparing the lag time, slope, and amplitude between the two pulses in single +/+ cells also shows that the majority of cells have a higher slope in their response to the second pulse (Fig. S6, *F–H*). These results suggest that while CXCL10 activation is random, the expression level and slope can be affected by the cell’s expression history.

To further confirm the stochasticity in CXCL10 expression, we compared IRF1, CXCL10, and CXCL9 expression in sibling cells. For siblings that divide between 11 h and 0 h before the addition of IFNγ, there is a weak positive correlation between sibling pairs for expression of each of IRF1, CXCL10, and CXCL9, and the correlation coefficient for CXCL10 is lower than that of IRF1 (Fig. S6, *I–K*). This lack of strong correlation between siblings, especially for CXCL10, is in accord with our repeated pulse experiment, supporting the intrinsic stochasticity in gene activation as described in our model.

## Discussion

In this work, we used endogenous fluorescent reporters to determine how three different IFNγ-inducible genes decode dynamic IFNγ stimulation in divergent ways. We found that macrophages express IRF1 strongly in response to low-concentration or transient IFNγ stimulation. In contrast, IFNγ stimulation must be higher in concentration or longer in duration for macrophages to express CXCL10 and CXCL9. Mechanistically, these different ways of decoding dynamic stimulus point to different mechanisms underlying expression of each gene. Previous work has shown that, in the absence of signal, chromatin at the IRF1 locus is ready for transcription, while the CXCL10 locus is slightly open but not yet transcriptionally active and the CXCL9 locus has repressive chromatin marks (82, 83, 84). These basal chromatin states align with the fast, homogenous IRF1 expression that we see, as well as the delayed expression of CXCL10 and the even more delayed expression of CXCL9. While our modeling provides a possible explanation of these delays due to the speed of chromatin remodeling, further experimental work is needed to elucidate the mechanisms of gene expression at each locus. For example, we find that treatment with A485, a p300 inhibitor, delays CXCL10 expression, pointing to p300 recruitment as important for the timing of CXCL10 expression (Fig. S7*A*).

We observe relatively uniform IRF1 expression among single cells but remarkable cell-to-cell variability in CXCL10 and CXCL9 expression. The heterogeneity seen for CXCL10 is particularly notable because CXCL10 is expressed relatively early and at a high level, which are characteristics generally associated with lower heterogeneity. Further, the existence of a subpopulation of low-to-nonresponding cells even when exposed to a constant saturating-concentration input is intriguing and cannot simply be attributed to slow chromatin kinetics. In contrast, the homogeneity in IRF1 expression and the heterogeneity in both amplitude and timing for CXCL9 expression are expected for a fast primary response gene and a delayed secondary response gene, respectively.

Our modeling suggests that the unique features of CXCL10 heterogeneity can be explained by slow, stochastic chromatin opening on a similar timescale to that of upstream signal adaptation (∼4 h). As a result, cells have a defined and limited time window in which to express CXCL10, and only a fraction of them will be able to open their chromatin in that window and initiate gene expression. In this way, the interplay of time scales for chromatin activation and upstream signal adaptation can enhance the heterogeneity in gene expression even for relatively fast-responding genes. This idea is further supported by our subsequent modeling and experimental analyses, showing that CXCL10 expression can be activated stochastically at each onset of stimulation with a similar distribution of high- and low-responding cells. The fast chromatin closing for CXCL10 in our model is also necessary to set this defined time window for CXCL10 expression. In contrast, while CXCL9 also has slow chromatin opening, in our model, it has slow chromatin closing, which leads to our result that CXCL9 is still expressed after the input signal has adapted. This shows how the interplay of gene expression parameters can lead to different gene expression behaviors in response to dynamic stimulation.

Our modeling also shows that the adaptation of upstream STAT1 signal is crucial for capturing the dynamics and heterogeneity of gene expression. Future work will clarify the mechanisms of this negative regulation and identify the specific molecular factors involved for each gene. In our modeling, we see that the cooperativity factor for production of the negative regulator’s mRNA is less than 1, which is unusual and could point to additional gene regulatory steps in the production of the negative regulator. It is also likely that there are additional general and gene-specific forms of negative regulation in this signaling pathway in addition to the common-to-all-genes adaptation that we model here, as negative regulation is a key feature of pro-inflammatory signaling. For example, it is known that there are specific mechanisms to negatively regulate CXCL10 expression (85, 86, 87). Including these may improve the model fitting even more. It is also important to note that while our data are consistent with this model of slow, stochastic chromatin opening, there are likely additional mechanistic elements involved in CXCL10 expression. We also note that while we assume TF in our model to be STAT1 and the three states to be closed, open-uninitiated, and open-initiated chromatin, there could be other interpretations of the model and further experiments would be required to confirm these assumptions.

Both the dynamics and heterogeneity of gene expression observed in this study could have important physiological relevance for macrophage functions. The differences in gene expression response to dynamic stimulus that we found may underlie the thresholds at which macrophages perform different tasks in response to pro-inflammatory stimulus. It is somewhat surprising that an upstream TF such as IRF1 saturates its expression at such a low concentration and duration of stimulation, but there are likely further levels of regulation affecting concentration and duration response for genes downstream of IRF1. The fast decay of IRF1 upon removal of stimulus could facilitate fast transcriptional changes when the stimulus is gone. In contrast to IRF1, CXCL10 and CXCL9 act as high-pass filters where they are only expressed in response to higher amounts of stimulus, and our modeling shows that this filtering can be partly explained by the slow chromatin opening for these genes. This may permit the macrophage to ascertain that there is a sufficient quantity of IFNγ to warrant recruiting T cells and further amplifying the immune response, so that the macrophage does not amplify the immune response unnecessarily. Further, the expression dynamics of different genes may enable a time-based coordination of various functions performed by macrophages in response to changing environmental signals. Future work could connect these dynamics to diseases where IFNγ signaling is perturbed. Future work connecting these findings to macrophage responses in the context of an *in vivo* immune response could also use macrophage models that more closely approximate macrophages *in vivo* or investigate how the response varies in different types of macrophages.

The heterogeneity in gene expression may also be functionally relevant for macrophages. We speculate that the wide distribution in gene expression may allow for more precise tuning of total population CXCL10 output. In addition, it may be more robust to perturbations than a system where there are stable populations of CXCL10-expressing and CXCL10-nonexpressing cells, as in the system seen here, a single cell can switch between expressing CXCL10 and not expressing CXCL10. This also allows for continued heterogeneity in response to repeated pulses, which may be functionally useful. Future work could engineer populations of macrophages to either respond as our cells do or to stably express CXCL10 at a specific level and investigate how this changes T cell recruitment and immune response progression *in vivo*. Heterogeneity in CXCL10 expression among genetically identical cells has been seen previously (21, 31), and there have been reports of heterogeneity in the expression of other secreted cytokines, which we also see here with CXCL9 (28, 88). In dendritic cells, different stimuli lead to different levels of heterogeneity in expression of the *Ifbn1* gene, which has been hypothesized to be a strategy to balance responsiveness and control (28). It would be interesting to investigate if high heterogeneity is a common feature of secreted cytokines in the immune system.

Our work also provides an extension from the single-cell work done on macrophage responses to primary infections to investigate how macrophages respond to cytokines that they experience in the middle of an immune response rather than at the beginning. As one point of comparison, we see that under conditions of constant IFNγ, IRF1 levels oscillate in both our experiments and our modeling. This is reminiscent of the NFκB oscillations that are observed in response to certain stimuli and suggests that more work should be done on oscillations of pro-inflammatory TFs to investigate if this is a more general phenomenon.

## Experimental procedures

### Cell culture

RAW 264.7 cells were ordered from ATCC (cat # TIB-71) and cultured in Dulbecco’s Modified Eagle Medium (DMEM) (Cytiva HyClone # SH30022FS) supplemented with 4500 mg/L glucose, 4.0 mM L-glutamine, 10% fetal bovine serum, and 1% penicillin/streptomycin. Cells were cultured at 37 °C, 5% CO2, and 90% humidity.

### IFNγ and A485 treatment

Cells were treated with IFNγ (Prospec Bio #CYT-358) at the concentrations stated in the text; if not stated, then the concentration is 10 ng/ml, which corresponds to 100 U/ml. For A485 treatment, cells were treated with 10 μM of A485 (Tocris #6387) for 2 h before addition of IFNγ.

### Cell line construction

We used CRISPR/Cas9 genome editing to tag genes by knocking in fluorescent proteins at their endogenous loci. We designed guide RNAs (gRNAs) for CRISPR/Cas9 genome editing using an online CRISPR tool (http://crispor.tefor.net/). We ordered three guides from Eurofins genomics and cloned them into pSpCas9(BB)-2A-Puro (Addgene #48139) plasmids using a one-step restriction-ligation protocol (89). To test the cutting efficiency of the gRNAs, we transiently transfected the gRNA plasmids into NIH3T3 cells using Lipofectamine 2000 at a 1:3 Lipofectamine:DNA ratio. Transfected NIH3T3 cells were selected with puromycin (1 μg/ml) for 2 days and then grown out to a confluent 10-cm plate (2–3 days). We extracted genomic DNA from these cells, amplified the 1kb region around the cut site using PCR, and sent the PCR amplicon for Sanger sequencing. The sequencing results were then analyzed with the Synthego ICE analysis tool (https://ice.synthego.com/) and we chose the gRNA with the best cutting efficiency.

We designed donor plasmids to have ∼1kb of homology on each side of fluorescent protein insertion site and used primer tails to synonymously mutate either the PAM site or the gRNA recognition sequence so that the knock-in allele cannot be edited again. We used PCR to amplify these homology arms from the genomic DNA and to amplify the fluorescent proteins from plasmids (SYFP2 from pSYFP2-C1 Addgene #22878, mCerulean and mCherry from existing plasmids in the lab). Donor plasmids were assembled in a pUC19 backbone using Gibson assembly and confirmed by Sanger sequencing. A flexible linker sequence (amino acid sequence GDGAGLIN) was used between IRF1 and the SYFP2 tag, and a T2A sequence was used between the protein and fluorescent tag for CXCL10 and CXCL9 (70). This T2A sequence acts as a translational skip site so that the endogenous chemokine can be secreted into the media and the fluorescent protein can be retained in the cell and so measured. We added an SV40-NLS tag to the fluorescent proteins driven by CXCL10 and CXCL9 so that we could measure fluorescence intensity in the nucleus. IRF1 and CXCL9 are tagged on the C-terminal end, and CXCL10 is tagged on the N-terminal end. The nuclear marker consists of an NLS-iRFP (90) driven by a constitutive EF1alpha promoter (gift from Jan Soroczynski) inserted into the Tigre locus (70). This nuclear marker greatly facilitates cell segmentation and tracking. Plasmids were purified for transfection using a Macherey-Nagel NucleoBond Xtra Midi EF kit (Macherey Nagel #740422.50). A full list of plasmids can be found below.

We used the Neon transfection system (Thermo Fisher Scientific) to transfect the cells. 2.5 × 10ˆ6 RAW 264.7 cells were used per electroporation, along with 15 μg of gRNA-Cas9 plasmid and 15 μg of donor plasmid in a 100 μL Neon tip using R buffer. The electroporation setting was 1680V, 20 ms, 1 pulse, after which the cells were plated into one well of a 6-well dish containing antibiotic-free DMEM. Three identical transfections were done per condition. The transfected cells were then grown to a confluent 10-cm plate (7–9 days depending on cell viability after transfection), induced with IFNγ, and sorted on an Aria Fusion cell sorter to select for cells expressing the newly knocked-in fluorescence protein. Cells were sorted into conditioned media with 20% fetal bovine serum in 96-well plates with one cell per well and then 12 clones were grown up and screened. The cell line used in this paper was created by knocking in the nuclear marker, then tagging IRF1, CXCL10, and CXCL9 sequentially and growing up single clones after each knock-in.

**Table:** 

Plasmid	Function	gRNA target sequence
NHB0797 pUC19 Tigre EF1a-SV40NLS iRFP	Tigre nuclear marker donor plasmid	
NHB0795 pSp-Cas9-2A-GFP Tigre gRNA 2	Tigre gRNA plasmid	ACAGAAAACATCCCAAAGTT
NHB0866 pUC19 IRF1-mVenus Cterm donor	IRF1 donor plasmid	
NHB0842 pSpCas9-2A-puro IRF1 gRNA 4	IRF1 gRNA plasmid	GACCCAAACTATGGTGCACA
NHB0922 pUC19 NLS-mCerulean-T2A-CXCL10 donor	CXCL10 donor plasmid	
NHB0881 pSpCas9-2A-puro CXCL10 gRNA 4	CXCL10 gRNA plasmid	CTGATGGGGAGGCTTCCGGA
NHB1049 pUC19 mCh-NLS-T2A-CXCL9 donor	CXCL9 donor plasmid	
NHB0916 pSpCas9-2A-puro CXCL9 gRNA 2	CXCL9 gRNA plasmid	GTTCTATTGAGTCACTGTGT

### Cell line screening

The 12 clones that were screened were first imaged on our microscope for fluorescence induction after IFNγ stimulation, with the goal being to choose a clone that was representative of the majority of the clones and had fluorescence matching what is known about the induction of that gene. The insertion region was also amplified by PCR and sequenced to identify any mutations that are present at that locus, and a clone was chosen that either had no mutation (CXCL10 and CXCL9, which are both homozygous tags) or else a mutation that did not affect protein function. The IRF1 tag is heterozygous. For IRF1, the tagged allele has perfect sequencing, and the nontagged allele has a twelve base-pair deletion that makes it so that, while the wild-type (WT) protein ends PSIQAIPCAP∗, the untagged IRF1 in our cells ends PSIQAP∗. However, this mutated allele produces the same level of IRF1 as a WT allele by Western blot and also induces IRF1 downstream genes to the same level as WT IRF1 by qPCR. There were no clones with IRF1 homozygously tagged, and this selected clone had the least affected WT allele. After each knock-in, we also induced cells from each clone with IFNγ and did qPCR for the tagged gene and several other IFNγ-induced genes to make sure that the induction matched WT induction and chose a clone where there was minimal difference in qPCR levels in the clone with tagged proteins versus the untagged WT cells.

### Microfluidic device fabrication

SYLGARD 184 silicone elastomer base was mixed with 10% of SYLGARD 184 silicone elastomer curing agent, degassed for 20 min, poured onto a custom wafer (72), degassed for 2 h, and then baked at 80 °C overnight. Individual polydimethylsiloxane chips were cut apart, had four holes punched in them, and were cleaned with ethanol, water, and tape. Coverslips were cleaned with heptane, methanol, and water, and then dried using compressed air. Chips were bound to the coverslip in a UVO binder and then baked at 80 °C overnight to bond permanently. Two individual chips were bound to each coverslip.

### Bulk plate–based assays

For bulk plate–based induction assays, used for RT-qPCR and Western blots, a confluent 10 cm or 6 cm plate of RAW 264.7 cells was induced with the stated concentration of IFNγ. The cells were then collected after the induction time, and pellets were frozen at −80 °C to be used in downstream RT-qPCR or Western blots.

### RT-qPCR

Total RNA was extracted from RAW 264.7 cells using Trizol. The RNA was then diluted to 100 ng/ml and converted to complementary DNA (cDNA) using a High Capacity cDNA Reverse Transcription Kit (applied biosystems, #4368814). qPCR was then done using PowerUp SYBR Green master mix (Thermo Fisher Scientific #A25776) on a QuantStudio 3 thermocycler. Reactions were performed in triplicate and compared to uninduced RAW 264.7 cells to calculate the fold-change.

Primer sequences are as follows.

**Table:** 

mHPRT1_q_F	AGCAGTACAGCCCCAAAATG
mHPRT1_q_R	ATCCAACAAAGTCTGGCCTGT
mIRF1_q_F	CAACCAAATCCCAGGGCTGA
mIRF1_q_R	TGCTTTGTATCGGCCTGTGT
mCXCL9_q_F	TGTGGAGTTCGAGGAACCCT
mCXCL9_q_R	AGTCCGGATCTAGGCAGGTT
mCXCL10_q_F	ATGACGGGCCAGTGAGAATG
mCXCL10_q_R	TCGTGGCAATGATCTCAACAC
mNOS2_q_F	TAGACCTCAACAGAGCCCTCA
mNOS2_q_R	CTCGAAGGTGAGCTGAACGA
mGBP1_q_F	CATCACAGTCAATGGGCCAC
mGBP1_q_R	CCAAAGTCAGGACTGCGTTC

### Western blot

Proteins were extracted from RAW 264.7 cell pellets using protein extraction buffer (50 mM Tris-HCl pH 8, 150 mM NaCl, 1 mM EDTA, 1% NP-40, supplemented with protease inhibitor cocktail (Sigma) and 1 mM phenylmethylsulfonyl fluoride) sitting on ice for 30 min. Western blots were run with a standard protocol. IRF1 antibody was D5E4 from Cell Signaling Technology.

### Immunofluorescence

RAW 264.7 cells were seeded onto coverslips in 24-well plates at a density of 4 × 10ˆ4 cells per well. After 24 h, the cells were induced with 10 ng/ml IFNγ at staggered times so that at the end of the experiment, there were cells that had been induced for 24, 8, 6, 4, 2, 1, 0.5, and 0 h. Cells were then washed with PBS, fixed in 4% paraformaldehyde for 15 min, and permeabilized with 0.5% TritonX-100 for 10 min. Cells were blocked for 30 min in 2% bovine serum albumin in 1× PBS, which was also used as the antibody diluent. STAT1 antibody D1K9Y (Cell Signaling Technology) was used at a concentration of 1:1000 and an incubation time of 2 h, followed by secondary antibody incubation (Cell Signaling anti-rabbit IgG F(ab’)2 fragment Alexa Fluor 488 conjugate) for 1 h. Slides were mounted in Prolong Diamond and cured overnight. Slides were imaged on a Nikon Eclipse Ti inverted microscope with a 500 ms exposure time for the far-red nuclear marker and a 300 ms exposure time on the GFP channel for STAT1.

### Imaging experiments

#### Plate

Cells from a confluent 10-cm plate were seeded 20 h before the experiment in 24-well plates at a density of 4 × 10ˆ4 cells/well. Immediately before imaging, cells were washed with PBS and 500 μL of phenol-red–free DMEM with 4500 mg/L glucose (Gibco cat# 31053028), 4.0 mM L-glutamine, 10% fetal bovine serum, and 1% penicillin/streptomycin was added to each well. The plate was then brought to microscope and imaged for 2 h before addition of stimulus and then for 48 h after. Stimulus was added with a micropipette while the plate remained on the microscope stage.

#### Microfluidic

Each chip on the coverslip was vacuumed for 3 min before cell loading. A confluent 10-cm plate of RAW 264.7 cells was washed with PBS, collected in DMEM, centrifuged at 200*g* for 3 min, resuspended in 3 ml of phenol-red–free DMEM, and loaded into the prevacuumed microfluidic device using vacuum loading to a density of ∼20 to 30 cells/trap. Each chip with cells loaded was attached to a syringe with phenol-red-free DMEM as well as a waste tubing and incubated in a standard tissue culture incubator at 37 °C, 5% CO2, with humidity for 20 h between seeding and setting up on the microscope to allow cells to adhere to the coverslip. All syringes have a manual turn-valve controlling output from the syringe into the attached PTFE tubing (Cole-Parmer EW-06417-21), and this switching was controlled manually. Immediately before setting up on the microscope, a second syringe containing phenol-red–free DMEM with 10 ng/ml IFNγ was attached to the chip but closed so that the cells continue to be in a no-IFNγ environment. The syringes and chip were then set up on the microscope, where the syringes sit outside the environmental chamber at 12 cm above the chip, and the waste lines also sit outside the chamber at 30 cm below the chip. The chip is in an environmental chamber controlling the temperature at 37 °C, 5% CO2, and humidity. The image ROI was set at 300 × 300 pixels, which corresponds to the size of one trap so that every image contains only one trap. Thirteen traps for each chip were chosen for imaging and were chosen to have a good number of nonclumped cells and to not image two adjacent traps (this minimizes phototoxicity). Images were taken for at least 2 h before onset of stimulation. At the onset of stimulation, the valve on the DMEM + IFNγ syringe was opened and the valve on the DMEM-only syringe was closed, and at the end of stimulation, the DMEM-only syringe was opened and the DMEM+IFNγ syringe was closed. Waste was collected in a 50-mL conical tube and measured to confirm that there was proper flow through the device during the experiment, which is about 0.5 ml/h. Experiments without the correct amount of flow were discarded.

#### All

Cells were imaged on a Nikon Eclipse Ti inverted microscope at 37 °C, 5% CO2, with humidity. Phase and iRFP nuclear marker images were taken every 10 min, while fluorescence images were taken every 60 min to minimize phototoxicity. Images were taken with a 20×/0.45 NA objective using the perfect focus setting and a Photometrics Evolve 512Delta EMCCD camera using Nikon Elements software (https://www.microscope.healthcare.nikon.com/products/software/nis-elements). Exposure times were 500 ms for iRFP, 500 ms for mCherry, 300 ms for SYFP2, and 100 ms for mCerulean.

### Image processing

Images were exported from the Nikon Elements software as multipage tifs and imported into CellProfiler. A CellProfiler pipeline was used to subtract the background using a rolling-ball algorithm. Nuclei were then identified from the iRFP nuclear marker images using the minimum cross entropy algorithm for the microfluidic chip and the Otsu algorithm for the 24-well plate, and a mask was made from these segmentations. The images taken from plate experiments and microfluidic experiments differ in their level of background and sharpness of the edges of the nuclei, which was why different segmentation algorithms worked best for each type of experiment and why we chose to use two different algorithms. Except in fitting our ODE model, the plate and microfluidic data are not compared against each other, and in the ODE modeling, there is an introduction of a scaling factor (see Modeling section) to make the plate and microfluidic values comparable. Fluorescence was quantified in each channel for each nucleus mask, and composite images were created with the object numbers overlaid on each fluorescence channel for manual confirmation of fluorescence and tracking. The images and a spreadsheet with the data were exported. Custom Matlab scripts were then used to organize the images into folders. We used u-track (91) implemented in Matlab to track the nuclei, and the tracking data were then converted into a format where each trace consists of the mean fluorescence intensity value for that cell at each timepoint. We then took only traces that are complete over the first 200 images of the experiment (∼33 h total, ∼31 h after IFNγ addition) and analyzed these further. All cells analyzed have strong trackable nuclear marker signal, indicating that they are alive for the duration of the experiment.

### Quantification and statistical analysis

#### Replicates

For concentration experiments, all concentrations were run in the same plate, and these plates were run in triplicate. One representative plate is used for all analysis shown. Each condition for plate experiments is a compilation of three wells of the 24-well plate and has 578 to 1016 cells. For microfluidic duration experiments, all conditions were run at least five times, with one chip on the coverslip running the experimental condition and the other chip receiving 4 h of stimulus as a control between days and chips. For each duration modulation condition, we combined all cells from 2 to 3 different runs of that condition and used that as the data for that condition. This results in each condition including 459 to 921 cells. The statistics on these combined datasets match the statistics for each individual run that makes up the dataset. These runs were chosen since they are representative of all runs and all had their paired 4-h control looking similar. For the microfluidic two-pulse experiment, this experiment was run three times and a representative run was chosen to show in these figures. The representative run chosen includes 367 cells.

#### General feature extraction

Features were extracted from all cells that had trajectories complete over the entire 33 h of the experiment. Maximum amplitude for all genes was calculated as the maximum amplitude over the first 24 h after IFNγ addition minus the mean of the two first images before IFNγ was added (which we suppose to be noise/basal gene expression and thus unaffected by IFNγ).

Lag time was calculated as the first time at which the fluorescence value was 1.5 times higher than that cell’s baseline level (defined as the mean of the first two images before IFNγ was added as well as the image right when IFNγ was added)†. In all cells, the values of these first three images (the two before IFNγ addition and the one right when IFNγ was added) were very similar.

Width for IRF1 was defined as the width at half of the maximum amplitude defined as the difference between the maximum value and the baseline expression. As CXCL10 and CXCL9 are transcriptional reporters and therefore do not represent endogenous decay rates, we do not calculate width for them.

Histogram boxplots were created using distributionPlot Jonas (2017). Violin Plots for plotting multiple distributions (distributionPlot.m) (https://www.mathworks.com/matlabcentral/fileexchange/23661-violin-plots-for-plotting-multiple-distributions-distributionplot-m), MATLAB Central File Exchange (Retrieved April 28, 2023) with bin edges held constant for all conditions in a plot, but each histogram scaled individually, so the shape of the histograms is comparable but individual bin height is not comparable. This was chosen because having individual bin height be comparable led to overlapping histograms between conditions, as in some cases there was one condition with the majority of its density in the smallest bin.

For the two-pulse experiment, maximum amplitude was taken to be the maximum amplitude from the time of IFNγ onset to 13 h after IFNγ onset (4 h of IFNγ + 9 h of time off) minus the expression at the time of IFNγ onset. This was chosen so that each pulse would be measured independently for itself and previous pulses would not contribute to measurements for later pulses.

#### CXCL10 feature extraction

The slope and pulse amplitude (used only in two-pulse experiment) for CXCL10 are extracted by fitting the initial CXCL10 rise (defined as the first 13 h after IFNγ addition) to y=(tanh(a⋅x+b))⋅c+d and defining the slope as a⋅c and the pulse amplitude as 2⋅c.

#### Sibling cells

Siblings are manually verified that they are in fact siblings, and their division time is defined as the first time when the cells are two separate cells. The sibling cells used in this analysis received 10 ng/ml IFNγ stimulus in a 24-well plate. In this experiment, cells were imaged for 11 h prior to addition of IFNγ and siblings were only used if they divided in that 11 h prior to IFNγ addition.

#### Cell cycle

Cells were imaged for 11 h prior to addition of IFNγ (addition of IFNγ being defined as time 0), and only cells that divided at some point during the experiment either before or after IFNγ addition were included in this analysis to define their cell cycle stage. Cells were placed into bins based on when they divided, with bin 1 being hours −11 to −7, bin 2 being hours −6 to −1, bin 3 being hours 0 to 4, bin 4 being hours 5 to 9, bin 5 being hours 10 to 16, and bin 6 being hours 17 to 22. This corresponds roughly to bin 1 being in G1/S when IFNγ was added, bin 2 being in G1, bin 3 being in G2, bin 4 being in S, bin 5 being in G1, and bin 6 being in G2. From observing cells that divided twice during the experiment, we see that cell cycle length for most cells is about 17 h but in some cells can be as long as 26 h.

#### Calculation of CV

The CV is defined as the SD of a distribution divided by its mean. We calculate general CV (in the main figures) by taking the CV of the expression level of all cells at each timepoint and plotting this result over time for all conditions. Calculation of CV for individual features was done by taking the SD of that feature in all cells divided by its mean.

#### Modeling

The block diagram of our nonadaptive model is shown in Figure 4*A*. In addition to the TF, for each gene of interest, it includes five species that are involved in eight reactions: mRNA (M), protein (P), and three possible states of the chromatin at the locus of each gene of interest: closed (C), open-uninitiated (OU), and open-initiated (OI). The transition rates among the chromatin states and the rates of synthesis and degradation of both mRNA and protein are shown in Figure 4*A*. We assume that mRNA synthesis can occur only when chromatin is in the open-initiated state. For simplicity, we also assume that the chromatin cannot close when it is in the initiated state. We chose to develop a three-state model based on the general understanding that both chromatin opening and transcription initiation require TF binding, and evidence in the literature that for IRF1, CXCL10, and CXCL9, IFNγ induction leads to STAT1 binding at their promoters (50, 51, 92, 93). The literature also shows that placement of H3K27ac active enhancer marks at associated enhancers requires STAT1 to be present, supporting our model that STAT1 is also required for the chromatin-opening step (51).

The concentration of the TF is a nonlinear function of the stimulus concentration [IFNγ],

(1) $$\left[\text{TF}\right]=\tanh\left(\alpha \left(\left[\text{IFN}\gamma \right]+K_{d}\right)\right)$$

Chromatin-opening rate and initiation rate are both proportional to [TF], while closing ($k_{2}$) and deinitiation ($k_{4}$) rates are constant.

In the generalized model that includes stimulus adaptation, we added two more species, regulatory mRNA (regM) and regulatory protein (regP), and five more reactions for the transcriptional negative feedback loop that suppresses the TF, see Figure 4*B*.

For deterministic modeling of the system without adaptation, we introduce mRNA and protein concentrations ([M] and [P], respectively) and the fraction of chromatin loci to be in each of the three states ([C], [OU], and [OI], respectively). The set of the ODEs for these variables reads as follows:

(2) $$\frac{d\left[C\right]}{dt}=k_{2}\;\left[OU\right]-k_{1}\left[TF\right]\left[C\right]$$

(3) $$\frac{d\left[OU\right]}{dt}=k_{1}\left[TF\right]\left[C\right]-k_{2}\left[OU\right]-k_{3}\left[TF\right]\left[OU\right]+k_{4}\left[OI\right]$$

(4) $$\frac{d\left[OI\right]}{dt}=k_{3}\left[TF\right]\left[OU\right]-k_{4}\left[OI\right]$$

(5) $$\frac{d\left[M\right]}{dt}=k_{5}\left[OI\right]-\delta_{M}\left[M\right]$$

(6) $$\frac{d\left[P\right]}{dt}=k_{6}\left[M\right]-\delta_{P}\left[P\right]$$

Because there are two chromatin loci for each gene, there is a conservation [C]+[OU]+[OI] = 2, and one of these differential equations can be eliminated.

The deterministic model with adaptation involves, in addition to the five equations above, the following two differential equations

(7) $$\frac{d\left[regM\right]}{dt}=a_{regM}\;\frac{{\left[TF\right]}^{c2}}{1+{\left[TF\right]}^{c2}}-\delta_{regM}\left[regM\right]$$

(8) $$\frac{d\left[regP\right]}{dt}=a_{regP}\left[regM\right]-\delta_{regP}\left[regP\right]$$

and the algebraic equation for the TF concentration that replaces (Equation 1):

(9) $$\left[TF\right]=\frac{\tanh\left(\alpha \left(\left[\text{IFN}\gamma \right]+\text{Kd}\right)\right)}{1+{\left(\frac{\left[reg_{P}\right]}{{reg}_{thr}}\right)}^{c1}}$$

We used these deterministic models for all three genes of interest (IRF1, CXCL10, and CXCL9). To fit the model, we used the data for the mean values of the fluorescence for each gene at each time in every concentration and duration condition. Since the conversion factor between the concentrations of proteins and the measured levels of fluorescence can differ between fluorescent proteins, before fitting, we rescaled the data such that the mean expression peaks at 40. The amplitude of fluorescence in the plate experiments (used for the concentration experiments) is higher than in the microfluidic device (used for duration experiments), so we scaled the microfluidic data based on the fact that the 10 ng/ml IFNγ plate condition should be equivalent to the 10 ng/ml constant IFNγ microfluidic condition. We calculated the scaling factor for each gene by dividing the maximum fluorescence in the 10 ng/ml IFNγ plate condition by the maximum fluorescence in the 10 ng/ml IFNγ constant microfluidic condition and then multiplied all microfluidic data values for that gene by this scaling factor. The scaling factor for IRF1 was 1.7757, for CXCL10 was 3.1105, and for CXCL9 was 3.3009. We used the MATLAB function fminsearchbnd for constrained optimization John D'Errico (2012), fminsearchbnd, fminsearchcon (https://www.mathworks.com/matlabcentral/fileexchange/8277-fminsearchbnd-fminsearchcon), MATLAB Central File Exchange (Retrieved April 13, 2023) to fit the models for each of the three proteins of interest (IRF1, CXCL10, CXCL9). The best fit parameters for each of these cases are given in Tables S2 and S3.

We chose a simplified model architecture for the adaptation circuit compared to the main gene expression model because we have very little data with which to fit parameters for the negative regulator and so using the full ODE model would involve introducing additional poorly constrained parameters and variables.

When we attempted to simplify our model by making only one step dependent on TF or by combining the two reversible TF-dependent steps into one reversible TF-dependent step, the model was not able to fit the dynamical CXCL10 and CXCL9 data as well. The two-step activation also provides the simplest way to have delayed expression with a smooth transition to a steep slope while also fitting the concentration- and duration-modulation responses. IRF1 could be modeled well by one reversible TF-dependent step because chromatin opening for IRF1 is very fast, but we chose to also model IRF1 with the two-step model to maintain the same model structure for all genes.

We note that there is a partial indeterminacy in the parameters obtained from fitting the deterministic model ‡ and parameters in Tables S2 and S3 were chosen to be in the range of plausible values and such that the stochastic simulations matched the observed variability.

For the stochastic simulations, we used the same species and reactions as in the deterministic models. We employed the direct Gillespie algorithm (75) to compute the numbers of molecules of each species as a function of time. We acknowledge that the Gillespie algorithm is only an approximate implementation of stochastic biochemical reactions in nonstationary environments (since we model experiments in which input TF changes with time), but since the characteristic reaction time in our model is much shorter than the timescale on which TF changes, this approximation is quite accurate. Since our fluorescence data contain arbitrary scaling factors with respect to the actual protein concentrations inside the cells, the rate parameters of the deterministic model also have arbitrary scaling factors. However, propensities in stochastic models determine not only the average number of molecules produced but also the level of random fluctuations around these averages. To determine the appropriate scaling for the stochastic model, we fit the deterministic model using a range of possible scaling factors and then used these parameters to perform the stochastic simulation and compare the distributions of maximal single-cell protein expression levels between the simulation and the experimental data. The best fit between these experimental and simulation distributions was obtained when the parameters were scaled to produce about 3 to 6 molecules of mRNA and 40 molecules of protein at the peak for each gene. After this scaling was fixed for the stochastic model, we used that set of parameters for the deterministic model as well, so that the deterministic models could be directly compared with stochastic simulations. This scaling means that the unit concentration in the deterministic model corresponds to one molecule per cell volume in the stochastic model. After generating multiple stochastic runs (∼1000 per each experimental condition), we performed statistical analysis of simulation data by computing the mean values, SDs, and the distributions of selected features of the runs (maximum amplitude, lag time, etc.).

## Data availability

Raw image files and fluorescence quantification are available from the Hao lab upon request.

## Supporting information

This article contains supporting information.

## Conflict of interest

The authors declare that they have no conflict of interest with the contents of this article.

## Footnotes

† For CXCL10, we can also extract lag time from these curves by fitting them to y = (tanh(a∗x+b))∗c+d and defining lag time as −(1+b)/a, which is the time when a line with slope a⋅c through the inflection point intersects with the baseline expression level. However, here do not use this calculation since it does not apply to IRF1 and CXCL9. Comparing these two methods of calculating lag time for CXCL10 provides very similar results. For the two-pulse experiment feature extraction, we consider responding cells to be cells that fit this tanh function well and nonresponding cells to fit it poorly.

‡ Given only a single species (fluorescent protein) for fitting, the dynamics for this species can be invariant with respect to suitable rescaling of pairs of reaction parameters, for example, by increasing translation rate and simultaneously decreasing transcription rate by the same factor. Using constraints on the expected rates of synthesis and degradation of mRNA and proteins, we were able to narrow down this degeneracy, but to completely eliminate it, more detailed multivariate data would be necessary.
