# Supplementary material for: Quantifying dynamic pro-inflammatory gene expression and heterogeneity in single macrophage cells
Source: J Biol Chem. 2023 Sep 9;299(10):105230. doi: 10.1016/j.jbc.2023.105230 (PMC10579967; doi:10.1016/j.jbc.2023.105230)
Supplement: Supplemental Figs. S1–S7 [file mmc1.docx]

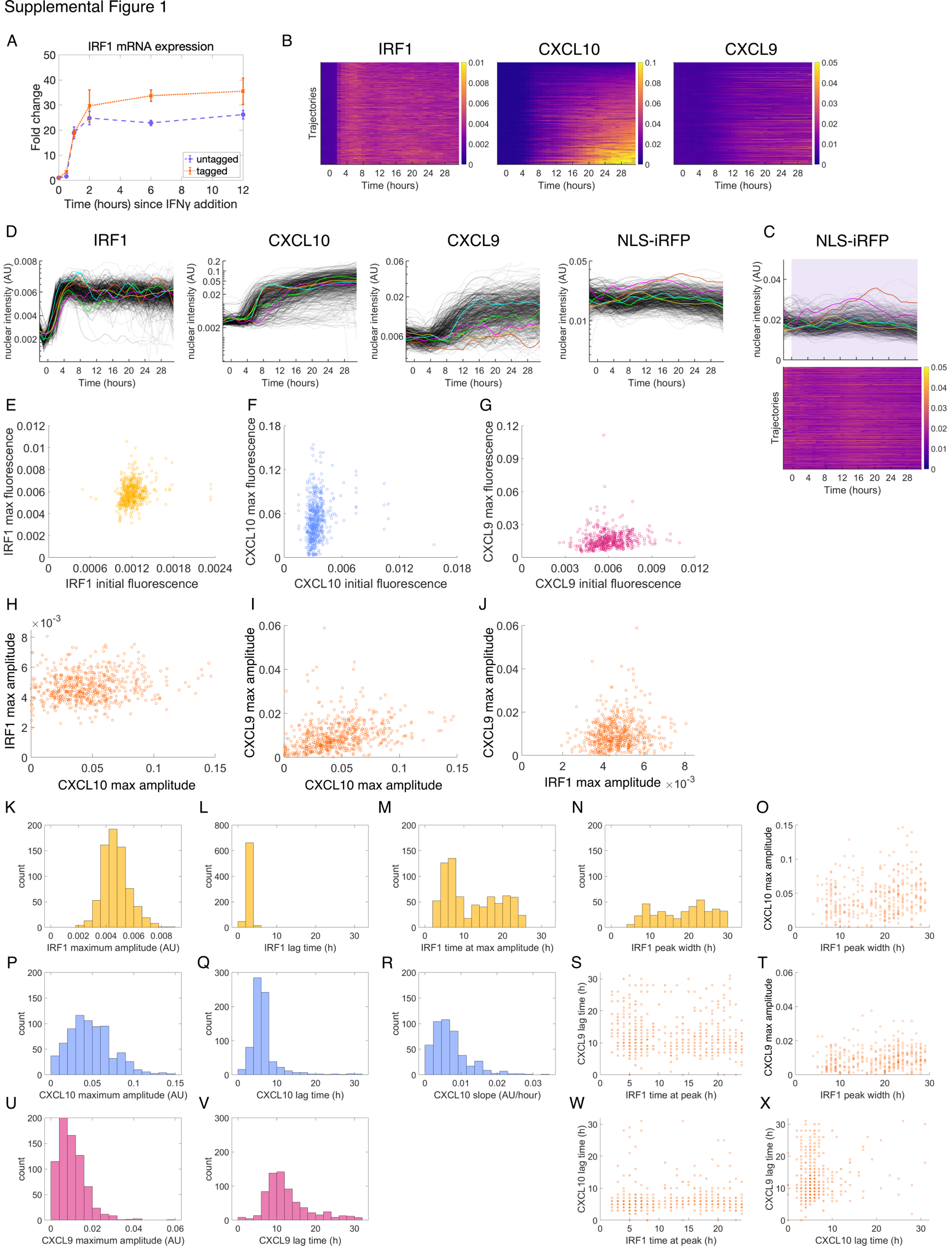


**Supplemental Figure 1**

A. qPCR data for the IRF1 gene from the untagged parent RAW 264.7 cell line and the quadruple-tagged cell line used in these experiments. Both cell lines were exposed to the indicated time period of 10 ng/mL IFNγ. B. Heatmaps of single-cell IRF1, CXCL10, and CXCL9 expression responses to 10 ng/mL IFNγ in a 24-well plate (same data as Figure 1C) sorted in the same order for all three genes. C. Nuclear marker NLS-iRFP expression in response to 10 ng/mL IFNγ stimulation in a 24-well plate, shown both as single-cell traces and as a heatmap with each row being one cell. D. IRF1, CXCL10, and CXCL9 gene expression responses to 10 ng/mL IFNγ in a 24-well plate, plotted on a log scale. Each grey line is a cell, with five exemplary traces in color. The data visualized is the same as Figure 1C. E-G. Scatterplots correlating the maximum amplitude of gene expression over the first 24 hours of IFNγ stimulation (the raw maximum amplitude, not the maximum amplitude with the baseline subtracted off that we use later for downstream analysis) with the initial fluorescence before IFNγ was added. H-J. Scatterplots correlating the maximum amplitude of the gene expression over the first 24 hours post-stimulation between different genes in the same cell. Each point represents one cell. K-N. Histograms of IRF1 features for cells exposed to 10 ng/mL IFNγ for 31 hours in a 24-well plate. P-R. Histograms of CXCL10 features for cells exposed to 10 ng/mL IFNγ for 31 hours in a 24-well plate. U-V. Histograms of CXCL9 features for cells exposed to 10 ng/mL IFNγ for 31 hours in a 24-well plate. O, S, T, W, X. Correlation of features of different genes in the same cells. For O, T each point represents one cell. For S, W, X each point represents one cell, and the opacity of the circle fill represents the number of cells at the same vertex.


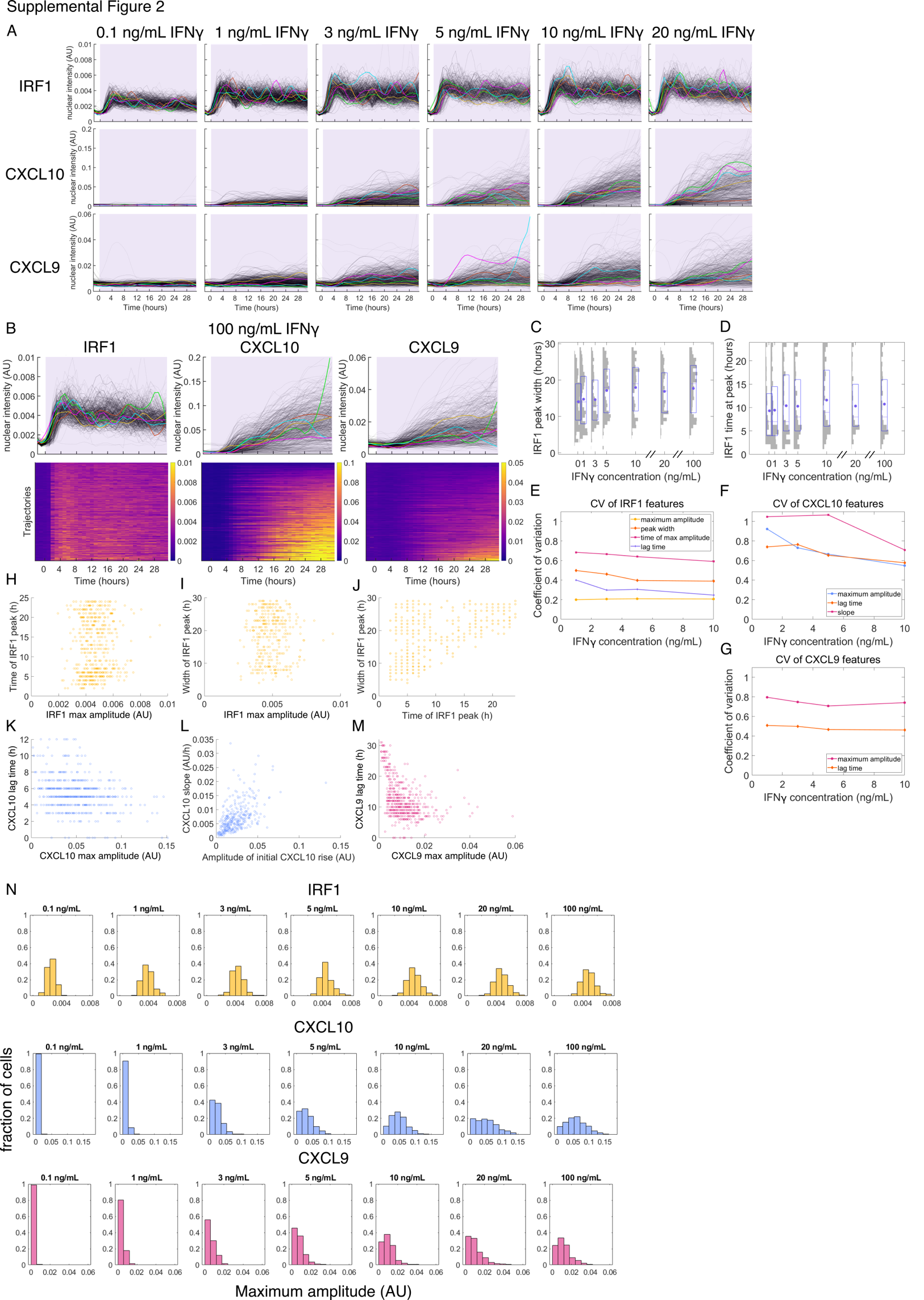


**Supplemental Figure 2**

A. IRF1, CXCL10, and CXCL9 gene expression responses to 1-20 ng/mL IFNγ in a 24-well plate. Same source data as Figure 2A-C but shown as single-cell traces. Each grey line is a cell, with 5 traces highlighted as examples. Purple shading indicates when the cells are exposed to IFNγ. B. IRF1, CXCL10, and CXCL9 gene expression responses to 100 ng/mL IFNγ in a 24-well plate. In the top row, each grey line is one cell, with five exemplary traces highlighted. Bottom row shows the same data as heatmaps with each row representing one cell, and each heatmap sorted by single-cell maximum value for that specific gene. Purple shading indicates when the cells are exposed to IFNγ. C-D. Histograms overlaid with box plots showing the IRF1 peak width (C) and IRF1 time at peak (D) in single cells for each IFNγ concentration. Purple dot is mean, middle purple line is median, and purple box is the 25^th^-75^th^ percentile. Grey shading shows the histogram distribution among single cells for each condition. E-G. Coefficient of variation for additional response features for IRF1 (E) CXCL10 (F) and CXCL9 (G). H-J. Scatterplots correlating features (H – amplitude vs peak time, I – amplitude vs peak width, J – peak time vs peak width) of IRF1 expression in response to 10 ng/mL IFNγ stimulus in a 24-well plate. K-L. Scatterplots correlating features (K – amplitude vs lag time, L – amplitude vs slope) of CXCL10 expression in response to 10 ng/mL IFNγ stimulation in a 24-well plate. M. Scatterplot correlating maximum fluorescence and lag time of the CXCL9 response to 10 ng/mL IFNγ stimulation in a 24-well plate. In the scatterplots, each dot is one cell and the opacity of the circle fill represents the number of cells at that vertex. N. Histograms showing the distribution of maximal expression amplitudes in single cells in response to 0.1-100 ng/mL IFNγ.


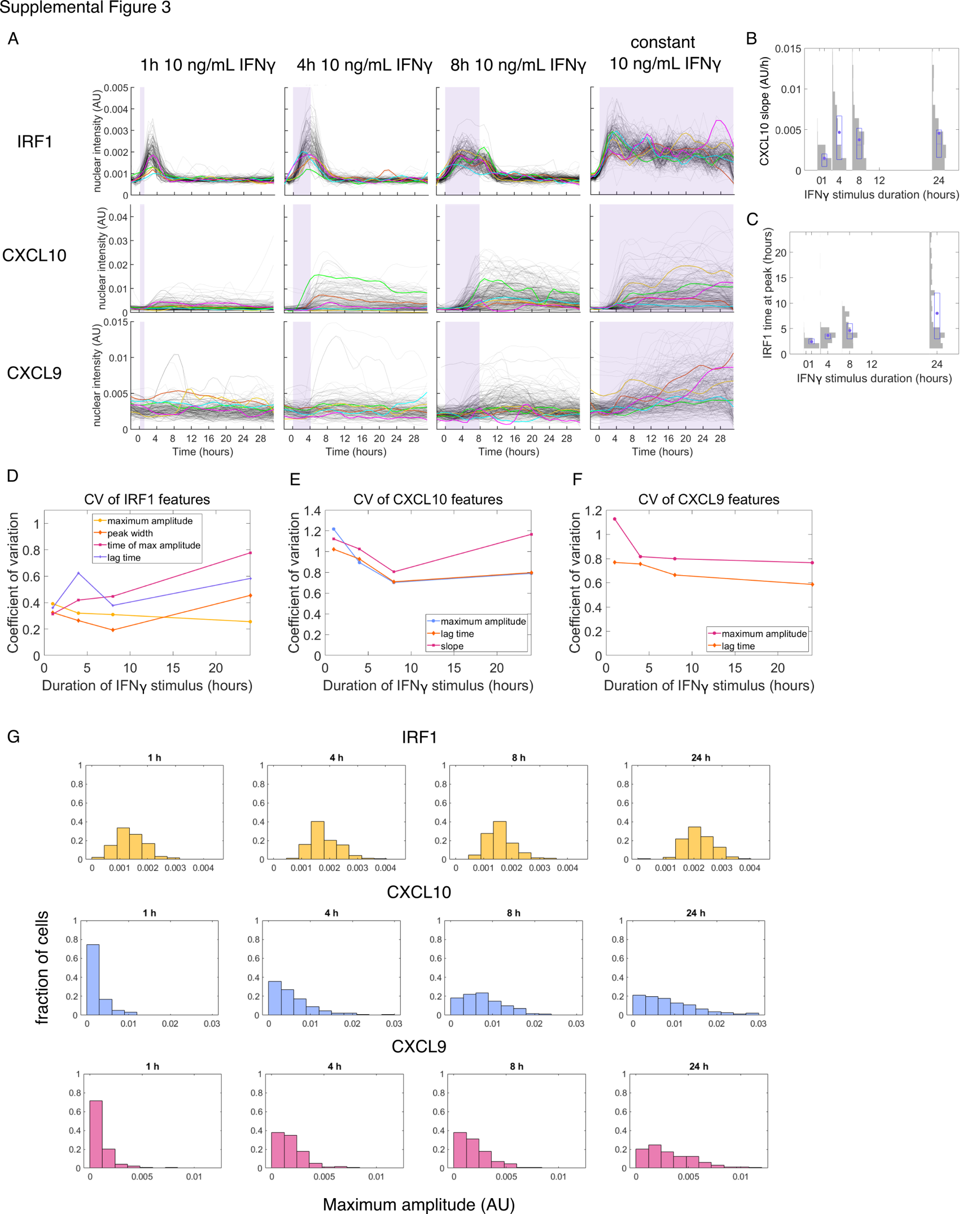


**Supplemental Figure 3**

A. IRF1, CXCL10, and CXCL9 gene expression responses to 1 h, 4 h, 8 h, and constant 10 ng/mL IFNγ stimulation in a microfluidic device. Same source data as Figure 3 A-C but shown as single-cell traces. Each grey line is a cell, with 5 traces highlighted as examples. B-C. Histograms overlaid with box plots showing the CXCL10 slope (B) and IRF1 time at peak (C) in single cells for each IFNγ duration. Purple dot is mean, middle purple line is median, and purple box is the 25^th^-75^th^ percentile. Grey shading shows the histogram distribution among single cells for each condition. D-F. Coefficient of variation for additional response features for IRF1 (D), CXCL10 (E) and CXCL9 (F). G. Histograms showing the distribution of maximal expression amplitudes in single cells in response to 1 h, 4 h, 8 h, and constant 10 ng/mL IFNγ in a microfluidic device.


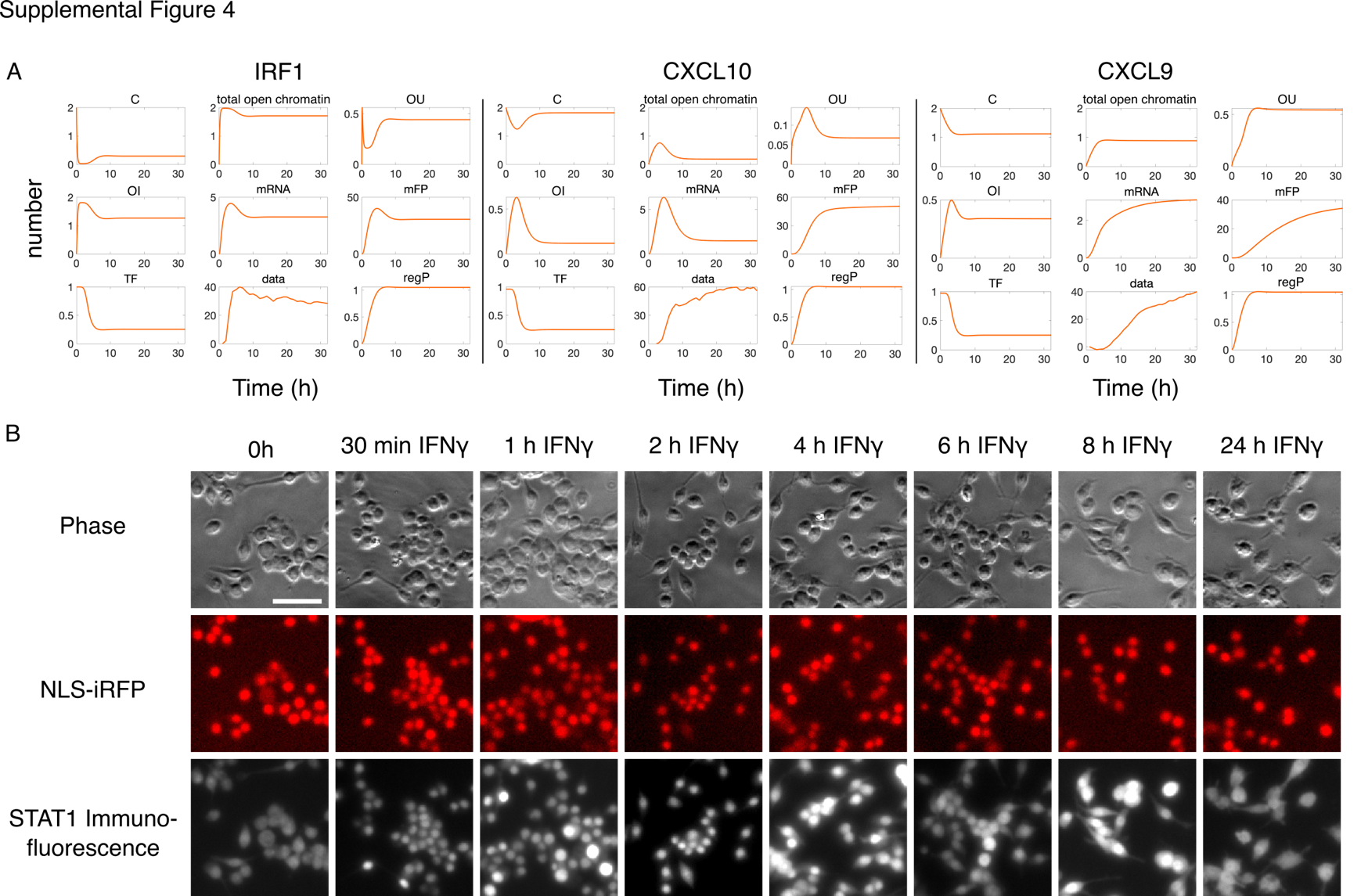


**Supplemental Figure 4**

A. ODE adaptation model intermediates for each of IRF1, CXCL10, and CXCL9 in the 10 ng/mL constant IFNγ stimulus condition. Names of reaction intermediates correspond to Fig 4A and B. B. Immunofluorescence for STAT1 in RAW 264.7 cells treated with 0-24 h of 10 ng/mL IFNγ. Scale bar on top left image represents 50 μm.


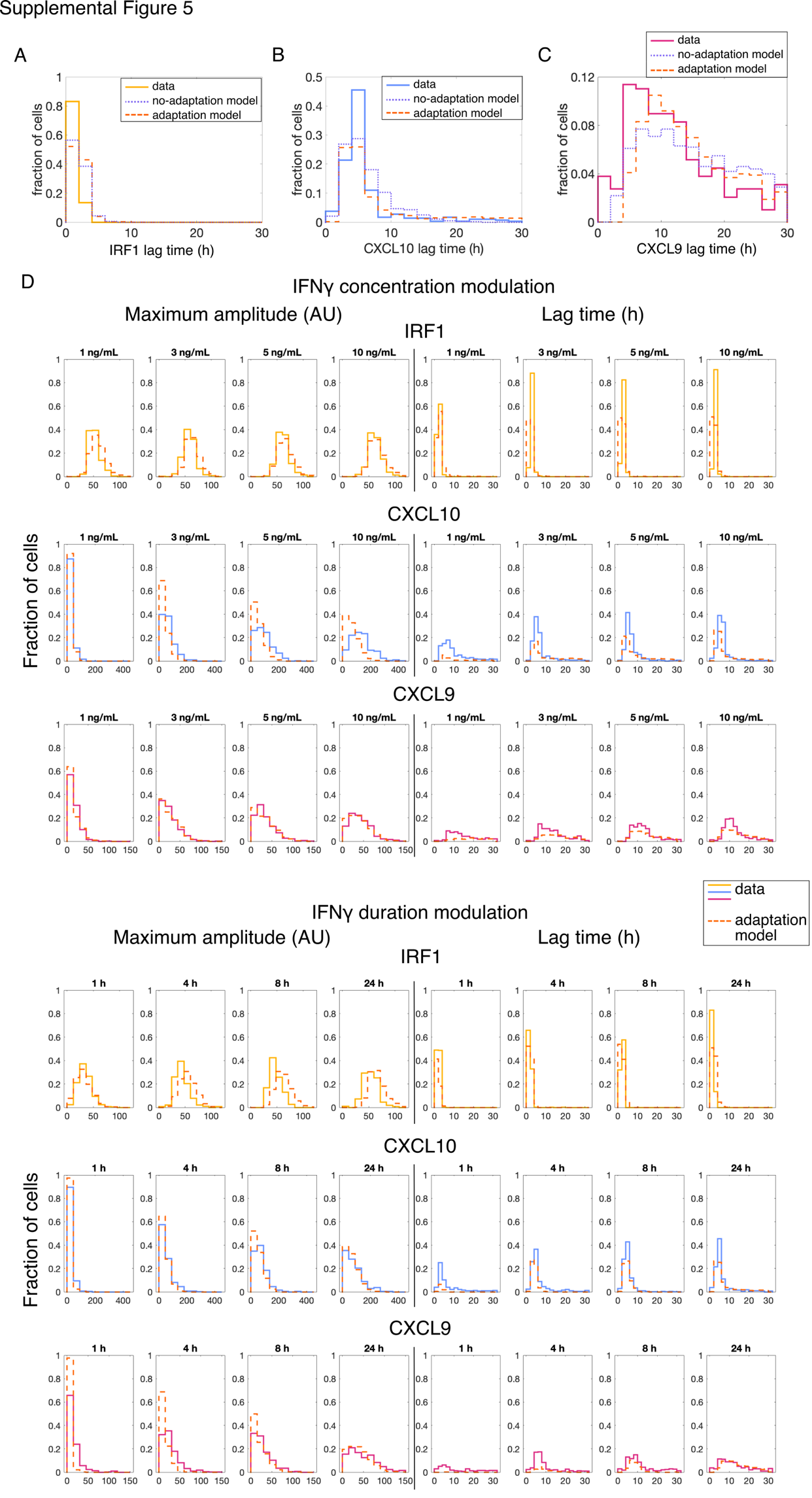


**Supplemental Figure 5**

A-C. Histograms showing distribution of IRF1 (A), CXCL10 (B), and CXCL9 (C) lag time both experimentally (solid line) and in the no-adaptation (purple dotted line) and adaptation (orange dashed line) stochastic models for constant stimulus of 10 ng/mL IFNγ. D. Histograms showing distribution of maximum amplitude and lag time for IRF1, CXCL10, and CXCL9 for various concentration and duration conditions in both the data (solid outline) and adaptation stochastic model (dashed orange line).


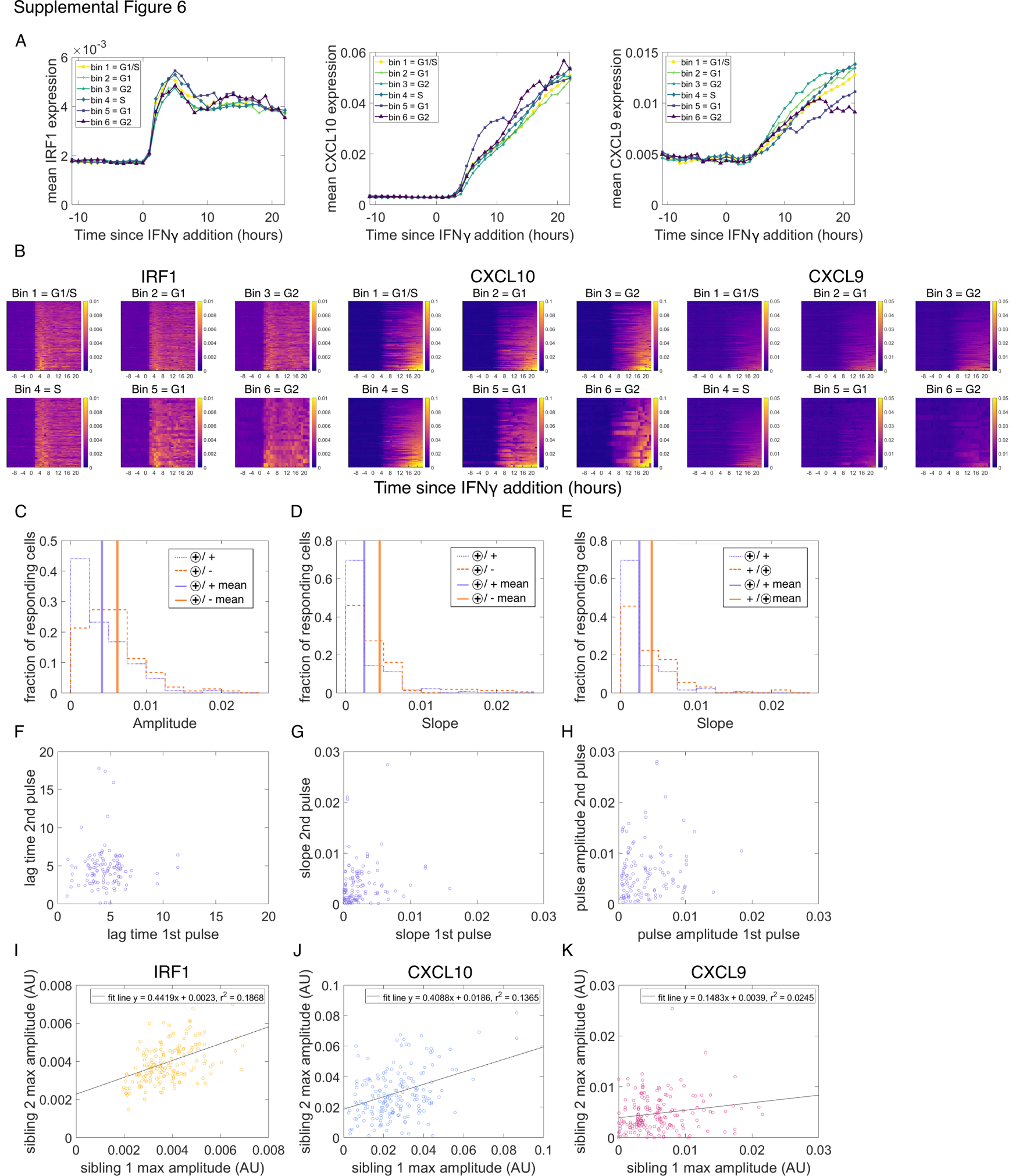


**Supplemental Figure 6**

A. Mean expression of IRF1, CXCL10, and CXCL9 in each cell cycle bin. B. Heatmaps showing expression of IRF1, CXCL10, and CXCL9 in each single cell, separated by cell cycle bin. Each row is one cell, and each heatmap is individually sorted by maximum expression. C and D. Histogram showing distribution of CXCL10 expression amplitude (C) and slope (D) in response to the first pulse of IFNγ between cells that respond to both pulses (+/+) and cells that only respond to the first pulse (+/-). The circled symbol shows the pulse that is being plotted. Solid lines show means of each distribution. Both the means (via t-test) and distributions (via KS test) are statistically significantly different at p<.01 both in the experiment shown and in all three replicates of this experiment. E. Histogram showing distribution of CXCL10 expression slope in response to both the first and second pulse of IFNγ in cells that respond to both pulses (+/+). Solid lines show means of each distribution. Both the means (via t-test) and distributions (via KS test) are statistically significantly different at p<.01 both in the experiment shown and in all three replicates of this experiment. F-H. Scatterplot of single cells that respond to both pulses (+/+) comparing the CXCL10 lag time (F), slope (G), and amplitude (H) in response to each pulse. I-K. Scatterplot showing the maximum IRF1 (I), CXCL10 (J), and CXCL9 (K) amplitude in the first 13 hours post-stimulation between sibling cells that divided in the 11 hours prior to stimulus onset.


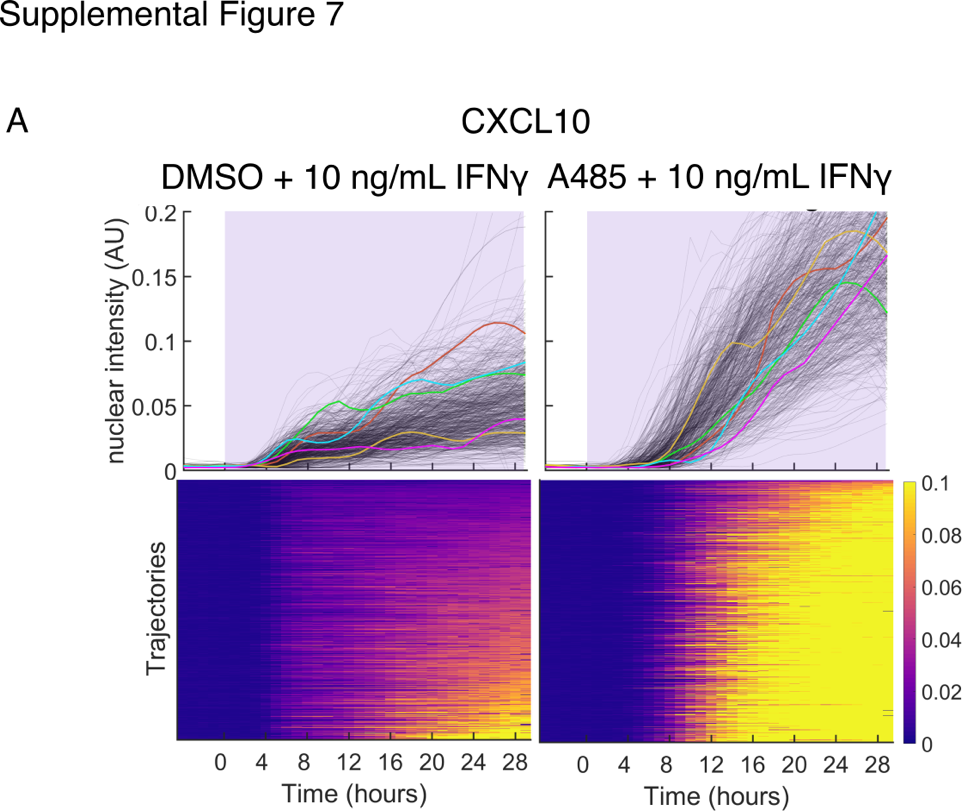


**Supplemental Figure 7**

A. CXCL10 expression in response to two hours of treatment with either DMSO or 10 μM of A485 followed by addition of 10 ng/mL IFNγ. Top row shows single-cell traces, each grey line is a cell, 5 traces are highlighted as examples. Purple shading shows when cells are exposed to IFNγ. Bottom row shows the same data as heatmaps with each row being a cell.
