# Supplementary material for: Quantifying dynamic pro-inflammatory gene expression and heterogeneity in single macrophage cells
Source: J Biol Chem. 2023 Sep 9;299(10):105230. doi: 10.1016/j.jbc.2023.105230 (PMC10579967; doi:10.1016/j.jbc.2023.105230)
Supplement: Supplemental Tables. S1–S4 [file mmc2.pdf]

Supplemental Table 1. Cell lines created in this work

Identifier	Name
NHM103	RAW 264.7 Tigre pEF1alpha-NLS-iRFP
NHM104	RAW 264.7 Tigre pEF1alpha-NLS-iRFP pIRF1-IRF1-SYFP2
NHM105	RAW 264.7 Tigre pEF1alpha-NLS-iRFP pIRF1-IRF1-SYFP2 pCXCL10-NLS-mCerulean-T2A-CXCL10
NHM106	RAW 264.7 Tigre pEF1alpha-NLS-iRFP pIRF1-IRF1-SYFP2 pCXCL10-NLS-mCerulean-T2A-CXCL10 pCXCL9-CXCL9-T2A-NLS-mCherry

All cell lines are available upon request from the Hao lab, and all tags are endogenous.

Supplemental Table 2. Parameters from fitting the deterministic ODE model both with and without adaptation to the data for each gene

	IRF1 no adaptation	IRF1 adaptation	CXCL10 no adaptation	CXCL10 adaptation	CXCL9 no adaptation	CXCL9 adaptation
k_1	10	6.2065	.9188	.3000	.1217	.18
k_2	3.0923	1.0454	2	2	1.5894	.0927
k_3	4.0999	10	.7269	8.1355	2.0036	10
k_4	.5884	.8913	.0435	1.1367	3.9950	4.0301
k_5	4.7614	2.9586	2.7842	7.7541	9.2615	1.2306
k_6	14.7298	10.7327	9.0105	1.3862	1.2439	1.5568
δ_M	1.9363	1.1467	.8501	.6265	.1055	.1329
δ_P	1.9363	1.1467	.8501	.0393	.1716	.1330
K_d	.0434	.0359	.025	.025	.1813	.0873
α	3.2926	4.2873	1.5	1.9256	1.9865	1.9710

Supplemental Table 3. Parameters used for adaptation of input IFNγ signal in deterministic ODE model

a_{regM}	3.8746
δ_{regM}	.6531
a_{regP}	.2710
δ_{regP}	.6530
reg_{thr}	.8792
$c1$	6
$c2$	.2144

Supplemental Table 4. Fraction of cells that do or do not express CXCL10 and CXCL9 in response to two four-hour pulses of IFNγ

By CXCL10 fitting:

Fraction of cells that respond to the first pulse	Out of all cells	.7493
	Out of cells that do respond to the second pulse	.6944
	Out of cells that don't respond to the second pulse	.8021
Fraction of cells that respond to the second pulse	Out of all cells	.4905
	Out of cells that do respond to the first pulse	.4545
	Out of cells that don't respond to the first pulse	.5978
Fraction of cells that respond to both pulses	Out of all cells	.3406
Fraction of all that respond to first * fraction of all that respond to second		.3675

By CXCL10 threshold:

Fraction of cells that respond to the first pulse	Out of all cells	.7629
	Out of cells that do respond to the second pulse	.6615
	Out of cells that don't respond to the second pulse	.8743
Fraction of cells that respond to the second pulse	Out of all cells	.5232
	Out of cells that do respond to the first pulse	.4536
	Out of cells that don't respond to the first pulse	.7471
Fraction of cells that respond to both pulses	Out of all cells	.3406
Fraction of all that respond to first * fraction of all that respond to second		.3991

By CXCL9 threshold:

Fraction of cells that respond to the first pulse	Out of all cells	.5493
	Out of cells that do respond to the second pulse	.3400
	Out of cells that don't respond to the second pulse	.5973
Fraction of cells that respond to the second pulse	Out of all cells	.1862
	Out of cells that do respond to the first pulse	.1214
	Out of cells that don't respond to the first pulse	.2727
Fraction of cells that respond to both pulses	Out of all cells	.0633
Fraction of all that respond to first * fraction of all that respond to second		.1023
